# Automatic Generation of a Mechanical Properties Question-Answering Data Set for Language Model Benchmarking: A Comparative Study of BERT, XLNet, and LLaMA Models

**DOI:** 10.1021/acs.jcim.5c02646

**Published:** 2026-03-27

**Authors:** Minglei Zhang, Jacqueline M. Cole

**Affiliations:** Ray Dolby Centre, Cavendish Laboratory, Department of Physics, 2152 University of Cambridge, J. J. Thomson Avenue, Cambridge CB3 0US. U.K.

## Abstract

Contextualized language models offer new opportunities for mining materials-science information from literature, but progress is limited by the absence of domain-specific question-answering (QA) data sets. This study addresses this by introducing MechQA, a data set of 202,068 pairs of questions and answers about mechanical properties that have been automatically distilled from 125,967 articles in the literature. Unlike small manually curated QA benchmarks or approaches that rely on domain-specific pretraining, MechQA provides a large-scale, automatically generated training resource derived directly from the primary literature. It covers five fundamental mechanical properties of materials: ultimate tensile strength, yield strength, fracture strength, Young’s modulus, and ductility. Manual evaluation of this data set confirmed its high quality (precision 83.76%, recall 89.09%, F1 score 86.34%). We apply MechQA to fine-tune three representative transformer models: two extractive models, BERT-base and XLNet-base, each with 110 M parameters, and a generative LLaMA-3.1-Instruct model with 8B parameters fine-tuned using low-rank adaptation (LoRA). The MechQA data set was partitioned into 181,722 training and 20,346 validation QA pairs for this application. On the validation set, domain-specific extractive models achieve strong Exact Match (EM) and F1 score performance (BERT: 78.03% EM/84.50% F1; XLNet: 78.21% EM/84.70% F1) with improved expected calibration error (ECE) of 7.98% and 6.25%, respectively, while the LLaMA-domain model achieves 80.48% EM/86.25% F1 with an ECE of 8.08%. Notably, the two extractive models exhibit competitive performance despite their significantly smaller parameter size compared to the LLaMA model. These results demonstrate that automatic QA data set generation, coupled with targeted fine-tuning, provides an effective data-centric method for domain adaptation of language models for materials science.

## Graphical abstract

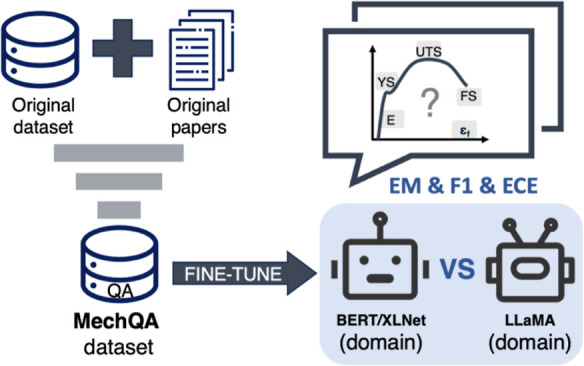

## Introduction

Data-driven materials discovery has become increasingly important, as modern materials research relies heavily on large and diverse data sets to guide design and optimization.^1–3^ However, a substantial amount of valuable materials knowledge remains hidden in unstructured forms within the scientific literature, limiting its direct use in data-driven workflows.^4,5^ Traditional rule-based information extraction approaches, limited by their rigidity, often struggle to capture the linguistic variability of such information at scale.^6^ In contrast, language models provide a more powerful and flexible framework for mining complex information from unstructured text, as they learn contextual representations that can handle terminology variation and generalize across various writing styles.^7,8^ While language models can be used in many ways, framing extraction as a question-answering (QA) task is particularly effective: it naturally mirrors how materials scientists query properties and avoids the rigid schema definitions that are required by other approaches.^9^ Bridging the gap between unstructured literature and structured data through QA-based language model approaches is therefore of great benefit for accelerating data-driven materials discovery.^10,11^

Despite their advantages, fully realizing the potential of language models in scientific domains presents substantial challenges. General-purpose language models often lack the specialized vocabulary and domain-specific knowledge that are needed for materials-science tasks.^12^ To address this, recent studies have explored domain adaptation for scientific QA through targeted fine-tuning, multiscale modeling, and model-merging strategies,^13^ alongside efforts in the domain-specific pretraining of language models such as SciBERT,^14^ MatSciBERT,^15^ BatteryBERT,^16^ OpticalBERT,^17^ and MechBERT,^18^ which demonstrate consistent improvements over general-purpose models. However, pretraining foundation models on domain corpora is computationally expensive, requiring extraordinary amounts of computation, time, and energy, with associated costs often reaching hundreds of millions of dollars.^8,19^ Such costs put training new foundation models beyond the reach of most academic research groups.^20^ These factors create a significant barrier for researchers with limited computational budgets and highlight the need for more efficient strategies to adapt language models to materials science.

Another major challenge hindering specialized applications of language models in materials science is the scarcity of sufficiently large and high-quality domain-specific QA data sets suitable for training language models.^21^ Data preparation is widely recognized as the most time-consuming component of artificial-intelligence (AI) projects, accounting for approximately 80% of overall effort.^22^ Moreover, limitations in the quality of data, such as inconsistencies, incompleteness, or extraction errors, when used to train models, can lead to unreliable model outputs and misleading scientific conclusions.^23^ This challenge is particularly acute in materials science, where expert-curated QA data sets remain scarce and are small in scale. For example, the recently introduced MaScQA benchmark contains only 650 materials-science questions,^24^ which can be valuable for evaluation but insufficient for training language models. In contrast, general QA benchmarks such as the Stanford Question Answering Data set (SQuAD) provide over 100,000 QA pairs about generic English queries.^25^ This order-of-magnitude disparity in generic versus domain-specific data availability has become a critical barrier to developing high-performing, domain-specific language models for materials property extraction.

Fortunately, a recently developed knowledge-distillation (KD)^7,26^ strategy enables the automatic construction of large and reliable QA data sets for materials-science domains, given the availability of an initial structured materials database. In this work, such a database was obtained using ChemDataExtractor,^27,28^ a materials-aware NLP toolkit designed to extract structured material-property relationships directly from scientific literature. ChemDataExtractor operates by identifying chemical entities using a combination of rule-based chemical grammars, machine-learning methods, and NLP techniques and language-model facilitated chemical named-entity recognition.^29^ In parallel, ChemDataExtractor also detects property values and associated units through regular expressions and linguistic analysis, and links materials to properties based on their co-occurrence and contextual relationships within sentences or paragraphs. The resulting output is a structured database containing material names, property types, numerical values, and units.

The KD method then transforms these ChemDataExtractor outputs into a QA data set by reverse-engineering the extracted material-property records into natural-language questions paired with their corresponding answers. Specifically, the original text contexts are treated as passages, and algorithmically generated questions query for materials, properties, or numerical values grounded in those passages. A key advantage of this reverse-engineering process is that it implicitly filters out NLP extraction errors: if a material-property association extracted by ChemDataExtractor does not actually appear in the source text, the KD algorithm cannot locate the corresponding context and automatically excludes that record. This self-cleaning mechanism ensures that only material-property relationships with verifiable textual evidence are retained, yielding a QA data set that is both large in scale and high in reliability. This approach is termed KD because such knowledge-rich QA data can then be used to fine-tune vanilla language models, enabling effective domain adaptation without requiring the knowledge to be encoded through expensive language model pretraining.^7,26^

Moreover, small language models (SLMs) have been shown to perform just as well as Large language models (LLMs) in this setup, because the size of a language model is no longer a critical requirement for pretraining their knowledge within them. This is important because it opens up the widespread use of SLMs when used in tandem with large domain-specific QA data sets. SLMs have many benefits: they are not dependent on the cloud to operate, so they offer a secure offline utility; they are far more energy-efficient than LLMs to train and run; they can be readily engineered as AI-agents to perform autonomous tasks in robotic procedures, e.g., within autonomous robotic laboratories of the future. This leads to an ultimate goal of developing data-driven materials discovery via autonomous design-to-device supply chains.

Building on this methodology, this study addresses three primary objectives. First, we develop a large-scale QA data set, MechQA, that is automatically distilled from materials-science literature. MechQA targets five fundamental mechanical properties of materials that are central to materials engineering: ultimate tensile strength (UTS), yield strength, fracture strength, Young’s modulus, and ductility. Second, we fine-tune and evaluate three QA models on the MechQA data set, including two extractive SLMs based on Bidirectional Encoder Representations from Transformers (BERT) and XLNet architectures that output numerical start and end token positions to identify answer spans, and a generative LLM based on the Large Language model Meta AI (LLaMA) 3.1 architecture that outputs answer tokens directly in natural language. The generative model is fine-tuned using low-rank adaptation (LoRA) to enable parameter-efficient training. Third, the study systematically compares the performance, robustness, and computational efficiency of these models, demonstrating that targeted fine-tuning on a materials-domain-specific data set yields high-performing specialist language models without incurring prohibitive pretraining costs. Figure 1 illustrates the overall workflow adopted in this study. The results show that a QA data set-driven distillation approach can effectively advance materials informatics, providing a practical means to overcome data scarcity and computational limitations in domain-specific AI development, and opening up new application areas for SLMs.

**Figure 1:**
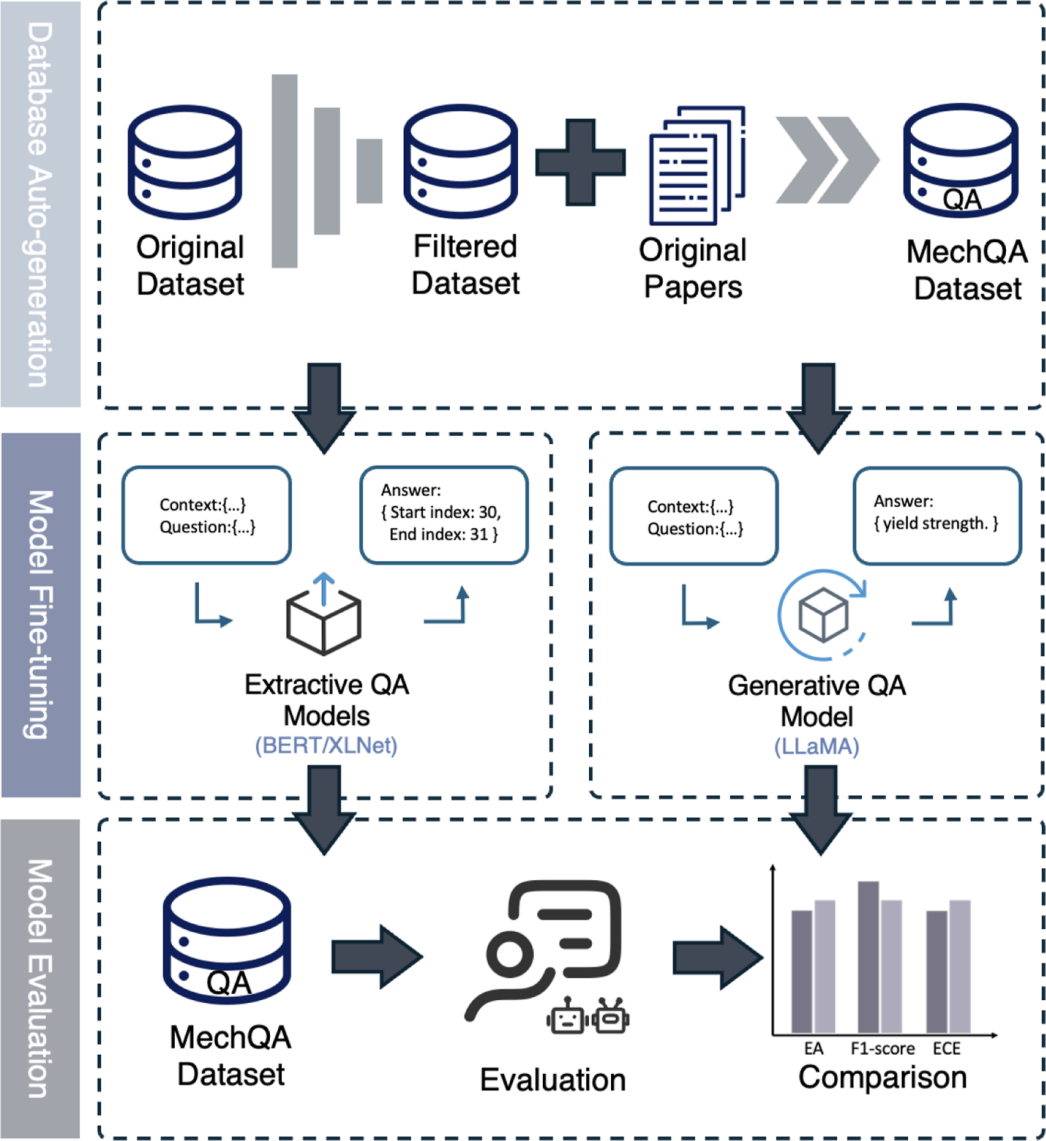
Overall framework of the data distillation and model fine-tuning adopted in this study.

## Methodology

### Establishing a Question-Answering Data Set about Mechanical Properties

Central to this study is the delivery of a domain-specific QA data set of mechanical properties.^30^ This data set was derived from a materials database about stress–strain engineering, which itself was derived from materials-science literature using the materials-aware NLP toolkit, ChemDataExtractor. The source literature consists of 125,966 scientific articles related to the mechanical properties of materials. The material names were mined together with their target properties, which included five fundamental stress–strain characteristics: UTS, yield strength, fracture strength, Young’s modulus, and ductility.

#### Creating a Filtered Material Data Set of Mechanical Properties

Each record in this material database comprises structured metadata such as compound name, property type, numerical value, measurement unit, and parsing method (text or table), along with bibliographic details of the papers from which the data record had been sourced, including their digital object identifier (DOI), article title, journal, author list, and publication date. Crucially, each data record retained contextual information about how the value was reported (e.g., whether it was parsed from the main text or a table), as for our purposes of autogenerating a mechanical QA, only text-parsed data records were retained in this study in order to ensure contextual integrity in subsequent QA tasks. Records with parsing inconsistencies or missing key fields were flagged and excluded to maintain data quality.

From the original corpus, 210,998 data records were parsed from text (excluding table parsing). However, certain data records contained multiple compound names associated with a single property. These cases were disaggregated using the explode function of the pandas library^31^ to generate a separate row for each compound while duplicating the associated mechanical-property data. This procedure yielded a curated data set of 325,449 records, providing a consistent structure for subsequent rule-based processing.

The list of unique material names extracted was initially quite noisy, containing 44,258 candidate names. Many were overly general or ambiguous terms, such as generic material classes (e.g., “steels”, “concrete”), elemental symbols (e.g., “Al”, “Co”), acronyms for microstructural regions (e.g., “HAZ” for heat-affected zone), or malformed tokens (e.g., stray fragments like “1-”, “SSSS”). To improve precision and reduce noise, a rigorous name normalization and filtering procedure was applied. This included the harmonization of text encoding, standardization of spelling variants, and exclusion of entries that did not contain alphanumeric characters. Terms matching curated lists of generic materials, isolated element symbols, welding or microstructural zone acronyms (e.g., HAZ, PMZ), or crystal lattice abbreviations (e.g., FCC, BCC, HCP) were excluded, and short fully capitalized acronyms were removed unless retained by an overriding domain-specific inclusion list. Additionally, a frequency-based heuristic removed highly frequent but nonspecific tokens (above the 95th percentile of corpus counts) that could not be unambiguously linked to a single material. Compositionally specific tokens (e.g., Ti–6Al–4V, Mg–Nd–Zn–Zr) were retained.

This QA data set-curation strategy had been designed to enhance data quality by mitigating the low precision of broad terms, resolving ambiguities arising from nonspecific identifiers, and reducing noise to ensure the reliability of the eventual QA data set for downstream tasks. In total, 4793 spurious or ambiguous compound entries were removed from the ChemDataExtractor-generated materials database about mechanical properties, yielding a final list of 39,465 compounds. After filtering, the data set contained 108,876 high-quality records of material-property relationships. The complete list of excluded compounds, together with their reasons for removal, are provided in the Supporting Information SI.

#### A Three-Stage Workflow to Auto-Generate a QA Data Set

A large-scale QA data set for training machine-learning models was automatically generated, using a three-stage algorithmic workflow that comprised: (1) full-text paper collection, (2) the extraction of relevant contextual information, and (3) the generation of structured question-answering pairs. This workflow is visualized in Figure 2.

1. Paper Collection. Papers were programmatically retrieved using DOIs via the application programming interfaces (APIs) of the Elsevier and Springer publishing houses. Each DOI was resolved to an XML representation of the full text and stored locally, with error handling implemented to bypass inaccessible documents and log problematic DOIs. In total, 125,967 full-text articles were successfully retrieved.
2. Extracting Relevant Contextual Information. Paragraph-level segmentation of each article was performed using ChemDataExtractor to preserve local contextual information. A multistage filtering procedure was then applied to identify paragraphs containing the three required elements: (i) compound name (matched with a case-insensitive regular expression that is tolerant of spacing and underscores), (ii) exact numerical value and its reported unit, and (iii) at least one mention of target mechanical property. For each property, a curated set of regular expressions captured both full names and commonly used abbreviations or symbols (e.g., “yield strength”, “Young’s modulus”, “UTS” for Ultimate Tensile Strength, “σ_*y*_” for yield stress, “E” for elastic modulus). Subsequently, a sentence-level scan was performed on these candidate paragraphs. Sentences containing predefined exclusion phrases indicative of comparative or range-based data (e.g., “from···to”, “range of”) were discarded to ensure the extraction of direct, unambiguous property assertions. For paragraphs containing multiple candidate sentences, a dynamic context window was constructed as the smallest contiguous block of sentences that encompass the property and value. This approach ensured sufficient context was retained while maintaining conciseness. Paragraphs exceeding 2000 characters were treated as excessively long. Such cases often arise from incorrect paragraph segmentation, where entire sections of text are parsed as a single block, or from paragraphs describing multiple materials or experimental conditions that could lead to spurious associations. In these cases, the context was automatically reduced to a smaller window surrounding the key sentence(s), thereby improving precision, reducing noise, and maintaining compatibility with the input length constraints of transformer-based QA models. This two-level filtering approach, consisting of paragraph-level selection followed by sentence-level refinement, ensured that each retained context established a precise and unambiguous association among the compound, property, and numerical value.

**Figure 2:**
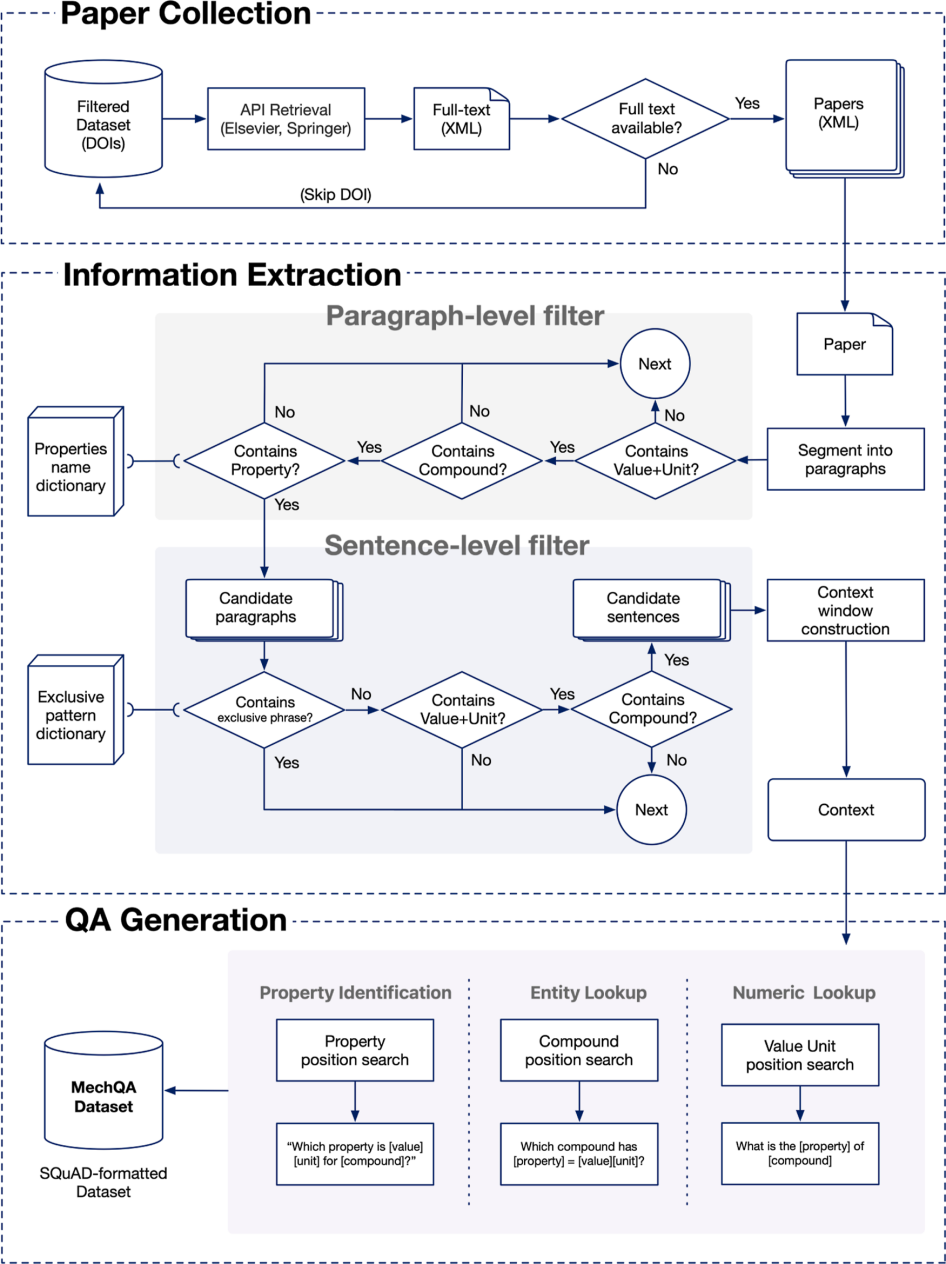
Algorithmic workflow for information extraction.

3. Generating Structured Question-Answering Pairs Each curated context was then used to generate a diverse set of QA pairs. Three complementary question types were constructed: (i) Numeric Lookup—querying the numerical value of a property for a given compound (e.g., What is the yield strength of [compound]?); (ii) Entity Lookup—querying the compound name given a property value (e.g., Which compound has a yield strength of [value]­[unit]?); and (iii) Property Identification—querying which property corresponds to a given value for a compound (e.g., According to the paragraph, which mechanical property is [value]­[unit] for [compound]?). For each QA pair, the answer was stored as an exact text span together with its character-level start index within the context, and the entire output was structured following the SQuAD format. This design ensured compatibility with standard extractive QA architectures and facilitated the direct fine-tuning of transformer-based language models.

To prevent data leakage, the QA data set was split by DOI into training and validation subsets using a 9:1 ratio, ensuring that no article contributed to both sets. The resulting QA data set comprises records that include the extracted context, encompassing the material name, mechanical property, numerical value, and unit, obtained from the original article, together with the corresponding generated QA pairs and their associated question type labels. Figure 3 provides a representative example of three MechQA data records.

**Figure 3:**
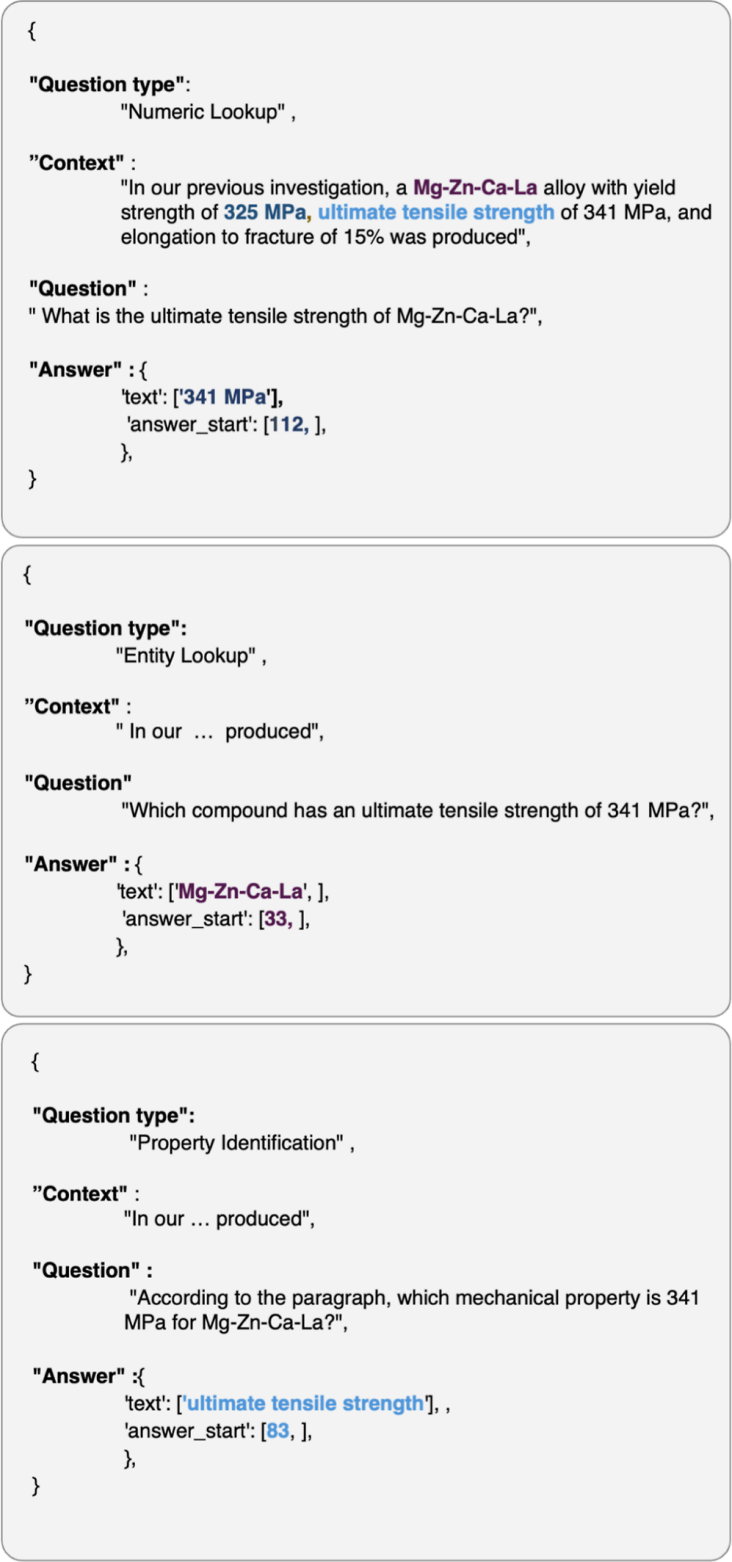
Representative examples of final QA records from the MechQA data set.

This largely automated, algorithmic approach to data set construction was designed to balance precision and recall. As the co-occurrence of a specific material, property, and value within the same paragraph was mandated, and contexts were filtered again at the sentence level, spurious material-value pairings that may occur in more naive text-mining processes are minimized. At the same time, comprehensive coverage of relevant statements was achieved by starting from a large literature corpus and applying broad regular-expression-based matching, including common aliases and symbols. The result is a high-quality QA data set grounded in peer-reviewed literature, suitable for training and evaluation of both extractive SLMs and generative LLMs.

### Fine-Tuning of Question-Answering Models

Three distinct transformer-based language models were fine-tuned on the curated MechQA data set to address the materials-science question-answering task: two extractive SLMs based on BERT^32^ and XLNet,^33^ and a generative LLM based on LLaMA 3.1.^34^ BERT^32^ was selected as the primary extractive baseline because it remains a robust and reproducible benchmark for span-based QA tasks, and is widely adopted in material applications.^35^ To complement this baseline, XLNet^33^ was included to represent an alternative pretraining framework to the dominant BERT-based models, as it is widely recognized as a common foundation model architecture within this field.^35^ Although newer architectures, such as RoBERTa^36^ or DeBERTa,^37^ can yield incremental performance gains, their higher architectural complexity and close structural similarity to BERT make BERT and XLNet more appropriate representative models for this study. For the generative LLMs, LLaMA 3.1 ^34^ was adopted as an open-source LLM with strong instruction-following performance and tractable parameter-efficient fine-tuning (PEFT).^38^ Alternative open models (e.g., BLOOM^39^ and GPT-NeoX^40^) were considered, but these either required substantially greater computational resources, showed weaker instruction-following abilities without additional adaptation, or lacked the ecosystem maturity of LLaMA at the time of study.^41^ Together, BERT, XLNet, and LLaMA 3.1 provide a reproducible and resource-efficient framework for comparing extractive and generative QA models in materials-science QA tasks.

#### BERT and XLNet for Extractive QA Tasks

BERT^32^ has a bidirectional transformer-based architecture that has been pretrained on large-scale corpora containing general English text, thereby enabling deep contextual representations of language. In order to make it materials-domain specific for mechanical properties extraction, a BERT model was fine-tuned on the curated MechQA data set. Following the approach of Li and Cole,^26^ the BERT-base-cased variant of this model architecture was selected to preserve case-sensitive information that is important in materials text (for example, chemical element symbols and acronyms are case-distinct); while the BERT-base distinction offered the 110 M parameters base-level BERT architecture for comparative purposes.

To provide a complementary language model architecture for the extractive QA task, XLNet^33^ was selected. XLNet employs a permutation-based autoregressive pretraining framework rather than the masked language modeling framework employed in BERT architectures, thereby reducing the discrepancy between pretraining and fine-tuning phases that arises from the use of masked tokens.^33^ In addition, XLNet incorporates the segment recurrence mechanism and relative positional encoding introduced in Transformer-XL, which facilitates better modeling of long-range dependencies compared to BERT’s absolute positional embeddings.^42^

The MechQA data set was converted into a SQuAD-style JSON format to enable integration with standard extractive QA fine-tuning pipelines. Each question–context pair was tokenized using the tokenizer that corresponds to the underlying model architecture: WordPiece^43^ for BERT and SentencePiece^44^ for XLNet. Both tokenizers segment rare or complex terms into subword units, ensuring that domain-specific expressions, such as alloy designations (e.g., Ti–6Al–4V) and measurement units (e.g., GPa), were represented without being treated as out-of-vocabulary. The tokenized question and context were concatenated and formatted using the model-specific special tokens required by each architecture.

Fine-tuning was formulated as a span-extraction task, in which the model predicts the start and end token indices of the answer span within the provided context. This formulation constrains predictions to spans explicitly present in the source text, thereby enhancing interpretability and ensuring consistency with the underlying literature. Both models were fine-tuned using the same data set and evaluation protocol. Hyperparameters were optimized for BERT, and the resulting configuration was subsequently applied to XLNet to enable a controlled comparison across model architectures, following a similar approach to that adopted by Yang et al.^33^

#### LLaMA 3.1 with LoRA for Generative QA Tasks

While newer LLaMA family models have been released since this study began, LLaMA version 3.1 represented the most stable and widely benchmarked configuration during testing. The LLaMA-3.1–8B-Instruct model was selected as a representative generative LLM for instruction-following question answering. Each QA pair from the MechQA data set was reformatted into a prompt-response format that is compatible with the model chat interface. Specifically, each instance included the extracted paragraph as context, followed by a natural-language prompt that had been derived from the question template (e.g., “Use the following paragraph to answer the question at the end. Only output the answer. Paragraph: [context] Question: [question]”). The corresponding answer from the data record served as the expected model response (e.g., “[answer.text]”). Input sequences were tokenized with the native tokenizer with additional special tokens to mark the start of the user query and the start of the assistant response. This format conversion allowed the model to be fine-tuned in a fully generative setting, where the task was to produce the correct answer text given the prompt.

To fine-tune LLaMA on our task within limited GPU memory, parameter-efficient fine-tuning (PEFT) was employed using LoRA.^45^ This approach freezes the original model weights and introduces small trainable low-rank matrices, reducing the number of trainable parameters by orders of magnitude. For example, Hu et al.^45^ reported that LoRA can reduce the number of trainable parameters by 10,000 times for a GPT-3-sized model with minimal loss in performance. For LLaMA, adapters were applied to the query (Q), key (K), value (V), and output (O) weight matrices within the self-attention layers.

Memory requirements were further reduced by quantizing model weights to 4-bit precision using the bitsandbytes library following the QLoRA methodology.^46^ This approach compresses models to approximately one-quarter of the memory footprints of their FP16 counterparts while maintaining minimal performance degradation. Additionally, training was distributed using DeepSpeed Stage 2,^47^ which applies optimizer-state partitioning and gradient accumulation to reduce memory overhead and improve scalability on limited hardware. During training, loss was computed only over the response tokens of the assistant by masking the prompt portion of the user; this ensured that the model learned to generate correct answers rather than simply reproducing the prompt.

#### Hyperparameter Optimization

Hyperparameter optimization was conducted using Bayesian optimization with the Weights & Biases (W&B) sweep framework^48^ to systematically search for high-performing configurations. For the BERT model, sweeps explored batch size, learning rate, maximum input sequence length, and number of training epochs, using Exact Match (EM) score on the validation set as the optimization objective. The search space was guided by prior recommendations for extractive QA fine-tuning,^32,49^ which suggested learning rates between 2 × 10^–5^ and 8 × 10^–5^, sequence lengths of 256 to 512 tokens, and 2 to 4 epochs for stable convergence. In addition to these ranges, weight decay was tuned between 1 × 10^–6^ and 0.01, following best practice for regularization procedures in transformer-based architectures. Each trial fine-tuned the model on a 10% stratified subset of the training data and evaluated performance on a 10% subset of the validation data to reduce runtime while preserving distributional coverage. Fixed parameters included: an input stride of 128, and a maximum answer length of 30 tokens; while an AdamW optimizer was set for linear learning-rate scheduling, and mixed-precision training (FP16) was set for computational efficiency.

For the LLaMA model, hyperparameter sweeps were conducted following best practice for the parameter-efficient fine-tuning of LLMs.^45,46^ The search space covered LoRA rank (*r* = 8, 16, 32), scaling factor (α = 16, 32, 64), dropout probability (0.0 to 0.1), learning rate (1 × 10^–5^ to 5 × 10^–4^), and warmup steps (50 or 100). Training was performed with a maximum sequence length of 1024 tokens, per-device batch size of 2, and gradient accumulation of 8 (effective batch size of 16). These values were fixed based on data set context length and GPU memory constraints; they were therefore not included in the hyperparameter search space. All runs used DeepSpeed Stage 2 for memory-efficient distributed training^47^ and computed loss only over assistant tokens by masking user instructions.

Each optimization sweep was executed on a single compute node of the Argonne Leadership Computing Facility (ALCF) Polaris supercomputer, employing its four NVIDIA A100 GPUs. The BERT sweep completed in approximately 12 h, and the LLaMA-3.1 sweep in 20 h. A summary of the search space for the hyperparameters of both eventual models is provided in Table 1. The best-performing configuration from each sweep was then used to fine-tune the final models on the full data set.

**Table 1:** Summary of the Hyperparameter Search Space for Model Fine-Tuning

model	hyperparameter	explored range/values
BERT-base-cased	learning rate	log-uniform ∈[2 × 10^–5^,8 × 10^–5^]
	weight decay	log-uniform ∈[1 × 10^–6^,0.01]
	batch size	8, 16, 32
	max sequence length	256, 384, 512
	epochs	2, 3, 4
LLaMA-3.1–8B-instruct	learning rate	log-uniform ∈[1 × 10^–5^, 1 × 10^–4^]
	LoRA rank (r)	8, 16, 32
	LoRA scaling factor (α)	16, 32, 64
	LoRA dropout	0.05, 0.1
	warmup steps	50, 100

#### Model Evaluation and Comparison

The fine-tuned models were evaluated on the held-out validation set using standard SQuAD-style metrics: EM and token-level F1 scores.^7,25^ EM measures the percentage of predictions that exactly match the ground-truth answer string, whereas F1 scores, the harmonic mean of precision and recall, reward partial overlap. These metrics are widely adopted in QA evaluations because they provide interpretable measures of accuracy by distinguishing exact correctness from partial correctness, and they enable fair comparison between extractive and generative methods.^8^ Although generative models are not constrained to output spans that are given verbatim, prompt engineering was applied to restrict outputs to a fixed format, and EM and F1 scores were computed by comparing the predictions with the reference answers.

Alternative metrics such as BLEU or ROUGE were not used, as they emphasize n-gram overlap and are less sensitive to small numerical discrepancies.^50^ The latter is critical in materials-science QA tasks where even slight deviations in reported values (e.g., 450 MPa vs 500 MPa) may be scientifically significant. An EM score is a strict measure that is suitable for factoid questions, whereas an F1 score provides a graded measure for cases where the prediction contains most of the correct answer but omits or adds minor details. Together, these metrics quantify both exact correctness and the degree of overlap when answers are not perfectly correct.

Beyond predictive accuracy, the reliability of model confidence estimates is also important for practical deployment in materials informatics workflows. A well-calibrated model ensures that the predicted probabilities match the actual empirical frequency of the outcomes, which is helpful for downstream QA tasks.^51^ Calibration quality was quantified using the Expected Calibration Error (ECE) metric, which provides a scalar measure of the discrepancy between predicted confidence and accuracy, as defined in eq 1.^52^

1 $$ECE=\sum_{m=1}^{M}\frac{\left|B_{m}\right|}{n}\left|acc\left(B_{m}\right)-conf\left(B_{m}\right)\right|$$

here, *M* denotes the number of confidence bins (set to 10), with predictions partitioned into *M* equal-width intervals. *B*_*m*_ represents the set of predictions whose confidence scores fall into bin *m*, |*B*_*m*_| denotes the number of predictions in that bin, and *n* is the total number of predictions. acc­(*B*_*m*_) is the accuracy in bin *m*, and conf­(*B*_*m*_) is the average confidence in that bin. Accuracy is computed using the EM between the predicted and reference answers in this study.

Confidence scores were computed according to the underlying model architecture. For extractive QA models (e.g., BERT and XLNet), the answer score *z*_ans_ was defined as the sum of the logits of the start and end positions, *z*_start_ and *z*_end_, respectively, as shown in eq 2.^53^

2 $$z_{ans}=z_{start}+z_{end}$$

Normalized confidence scores were obtained by applying a softmax over the top-*K* candidate spans (set to 10).

For generative QA models (e.g., LLaMA), given an input question *X*, a candidate answer set *I* = {*Y*}_*K*_ was generated using the beam search method.^54^ For each candidate answer *Y* = (*y*_1_, ..., *y*_|*Y*|_), the unnormalized probability *P*′(*Y*|*X*) was calculated by eq 3.^55^

3 $$P^{\prime }\left(Y|X\right)=\prod_{i=1}^{\left|Y\right|}P\left(y_{i}|X,y_{<i}\right)$$

where, *y*_*i*_ denotes the *i*-th generated token and *y*_<*i*_ denotes the previous generated *i* – 1 tokens. The unnormalized probabilities of the candidate answers were then normalized over the candidate set *I*. Specifically, the normalized probability of the top-ranked candidate Ŷ was computed using eq 4.^55^

4 $$\mathrm{P̂}\left(Ŷ|X\right)=\frac{P^{\prime }\left(Ŷ|X\right)}{\sum_{Y^{\prime }\in I}P^{\prime }\left(Y^{\prime }|X\right)}$$

The normalized probability P̂(Ŷ|X) of the selected top candidate was used as the confidence score. The resulting confidence scores were subsequently used in the computation of ECE.

In addition to these overall scores, metrics were computed separately for each question-type category (Numeric Lookup, Entity Lookup, Property Identification) and each mechanical property (UTS, yield strength, fracture strength, Young’s modulus, and ductility), to assess model performance across different attributes. Three benchmark models were also evaluated for comparison, including: (a) BERT-SQuAD, a BERT-base-cased model fine-tuned on the general-English language SQuAD without exposure to materials-specific data; (b) XLNet-SQuAD, an XLNet-base model fine-tuned on SQuAD; and (c) LLaMA-3.1–8B-Instruct, evaluated without domain-specific fine-tuning and provided with the relevant context at inference time, representing a zero-shot generative benchmark. These benchmarks allowed the benefit of domain-specific training to be quantified by comparing models of similar scale that contain or lack materials-domain knowledge.

## Results and Discussion

### Characterization of the MechQA Data Set

The MechQA data set was analyzed to characterize its overall structure and quality, providing essential context for its application in model fine-tuning. The following sections analyze key aspects of its composition, linguistic properties, and the reliability of the automated extraction process.

#### MechQA Data Set Composition and Properties Distribution

The MechQA data set was examined to characterize its composition, which is important for interpreting model performance. The generation workflow produced 202,068 QA pairs (181,722 for training and 20,346 for validation), all grounded in the scientific literature. To the best of our knowledge, MechQA is currently among the largest domain-specific QA data sets that have been resourced from materials-science literature. The distribution of the five target mechanical properties in the MechQA data set reflects their prevalence in the source literature. Figure 4 shows the percentage breakdown and the absolute counts of each property in the training set compared to the validation set. UTS is the most prevalent property, accounting for about 42% of all data records, followed by yield strength at 35%. Ductility and Young’s modulus each constitute about 10–11% of the data set, while fracture strength is the rarest reported property at 2.4% of all data records in training (2.5% in validation). These proportions are detailed in Table 2.

**Figure 4:**
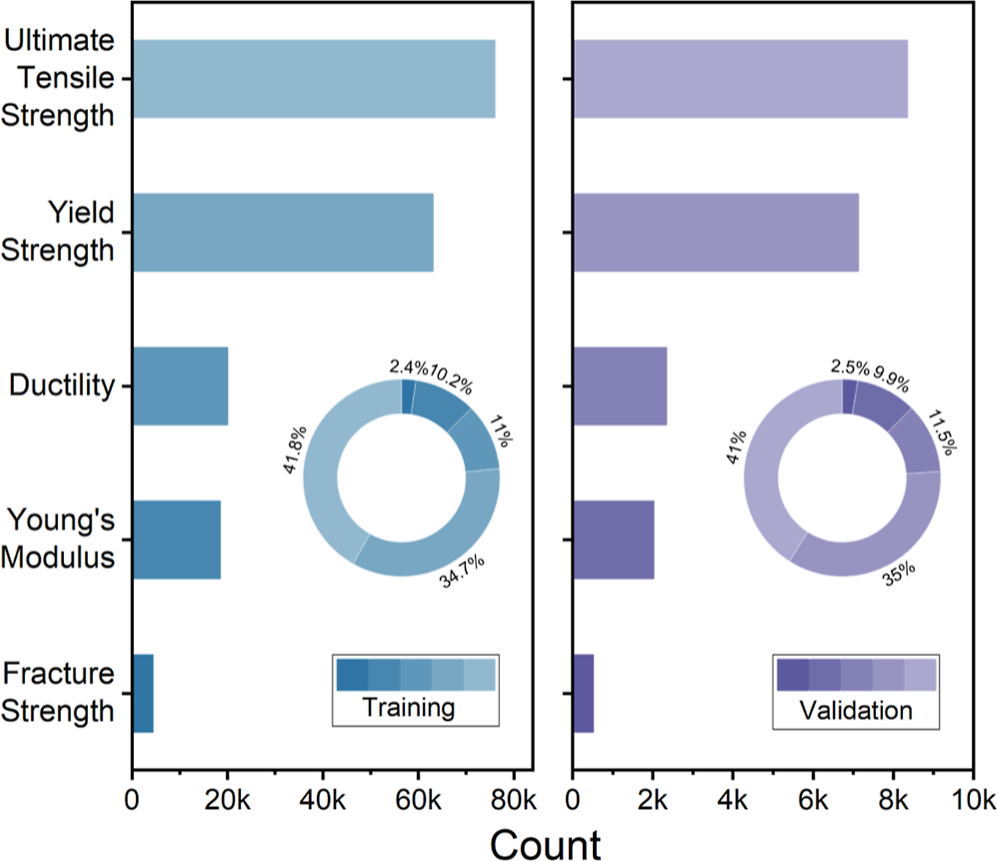
Distribution of mechanical properties in the MechQA data set: absolute counts of each data record type in the training set and validation set. Circular inset: percentage of data set record types in the training set and validation set.

**Table 2:** Distribution of Mechanical Property Records in the MechQA Dataset

property	training		validation	
	count	%	count	%
ultimate tensile strength	75,987	41.81	8343	41.01
yield strength	63,033	34.69	7125	35.02
ductility	19,929	10.91	2340	11.50
Young’s modulus	18,477	10.17	2022	9.94
fracture strength	4296	2.36	516	2.54
total	181,722		20,346	

This data imbalance arises naturally from human data-reporting prediction. Tensile and yield strengths are frequently reported in the literature for metals and alloys, whereas explicit fracture strength data appear less often. The automated data-extraction process preserved these trends. As a result, models trained on the MechQA data set may achieve higher accuracy on questions concerning UTS or yield strength due to the abundance of examples of associated QA pairs, while performance on fracture strength may be lower owing to the much more limited training data on this property characteristic. This illustrates the broader point that data set representativeness influences model behavior, though this issue can be mitigated through category-specific evaluation. Despite these data imbalances, the MechQA data set provides tens of thousands of QA examples across multiple properties, offering a strong basis for training language models and prompt engineering.

#### Contextual Characteristics of the MechQA Data Set

Beyond quantitative metrics, the textual features and contextual structure of the data set were analyzed to confirm its suitability for fine-tuning transformer architectures. Shown in Figure 5, the training set and the validation set have similar distributions, with a mean of 170.7 words for the training set and 170.4 words for the validation set. These lengths are well within typical limits for a BERT-base architecture, which can process 300–400 words comfortably within its 512-token capacity, and are short enough to be handled by LLaMA within a single prompt. This consistency reflects the data set design: by restricting contexts to the immediate sentences surrounding each property instance and truncating overly long paragraphs, the resulting passages provide concise yet sufficient information. Such compact contexts reduce the likelihood of irrelevant content or multiple candidate answers and avoid overly long sequences that could hinder training. In short, the MechQA contexts are both compact and complete, facilitating efficient model learning.

**Figure 5:**
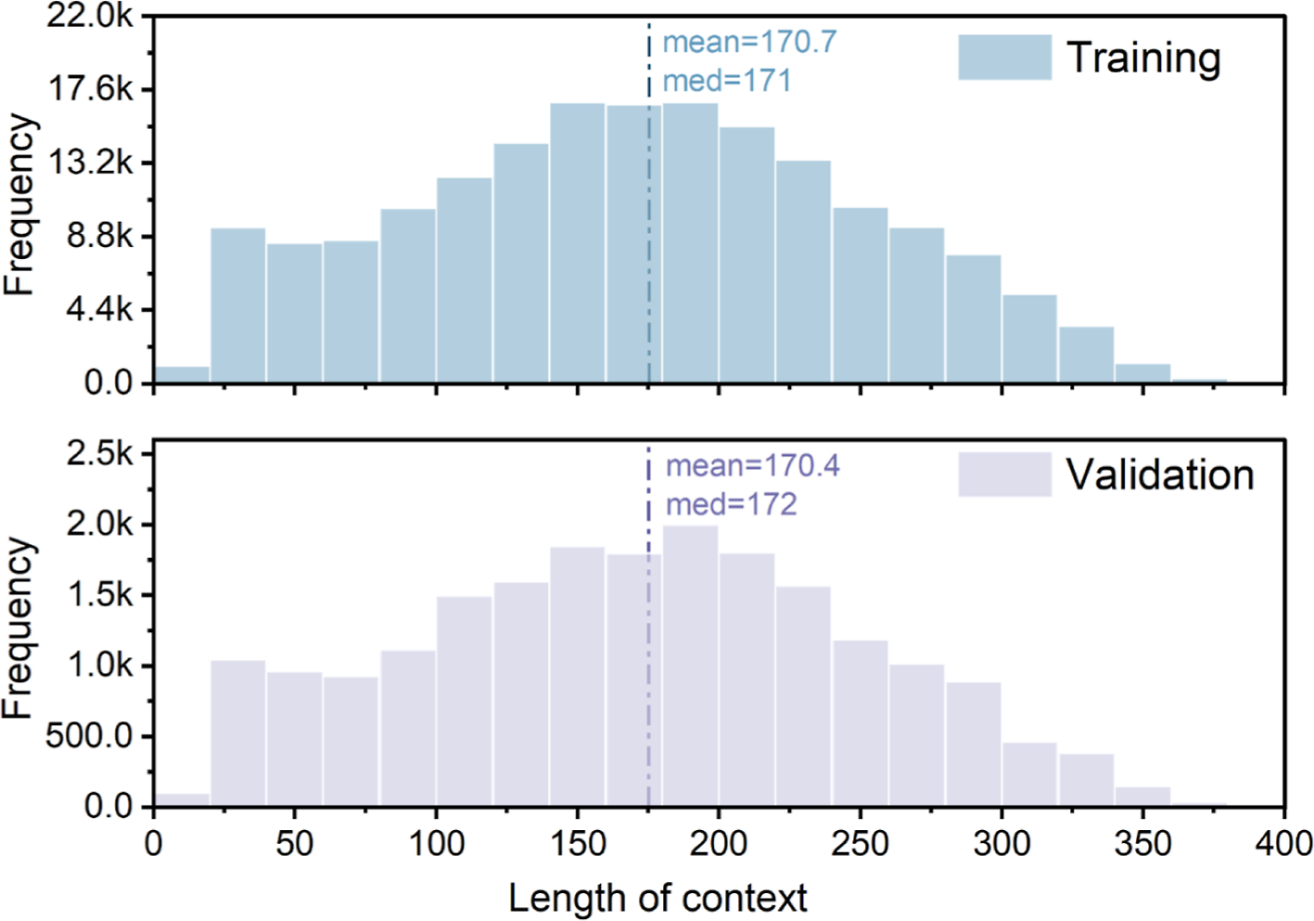
Distribution of context lengths (in word-count) for the training set and validation set.

#### Evaluation of the Workflow Used to Autogenerate the MechQA Data Set

The quality of the information-extraction workflow used to create the MechQA data set was assessed by performing a manual evaluation on a representative subset of the entire MechQA data set. 200 entries were randomly selected from the filtered data set and ran through the workflow to produce the subset. This is evaluated against a reference data set that is manually produced from the same 200 entries. For each entry, the verification process involved assessing whether or not the material, property, and value had been correctly extracted and were consistent with the surrounding context. In addition, the correspondence between the material and the associated property–value pair was examined, which is particularly critical for fine-tuning purposes. Precision, recall, and F1 score metrics were subsequently calculated for the evaluation of the extraction workflow, using this manually verified set as the ground truth. according to the standard formulae shown in eqs 5–7.

5 $$precision=\frac{TP}{TP+FP}$$

6 $$recall=\frac{TP}{TP+FN}$$

$$F1\;score=2\cdot \frac{precision\cdot recall}{precision+recall}$$ 7

where true positive (TP) refers to a correctly extracted record, false positive (FP) to an incorrectly extracted or erroneous record, and false negative (FN) to a relevant record in the reference data set that was not captured by the workflow.

As listed in Table 3, the information-extraction workflow achieved a high token-level F1 score of 86.34% on a manually annotated subset of the extracted MechQA data records, with a precision of 83.76% and a recall of 89.09%. These results represent a substantial improvement over the original data set, which showed an estimated precision of approximately 74% and an F1 score of 71%. Thus, precision was improved by nearly 9 percentage points while recall simultaneously increased. The higher precision demonstrates that the additional cleaning steps, such as the removal of ambiguous names, effectively reduced false positives and minimized noise in the MechQA data set. Importantly, this improvement was achieved without a major reduction in coverage, as recall remained high, ensuring that valid MechQA data records were not disproportionately discarded. Some of the remaining recall errors can be attributed to inconsistencies in compound nomenclature; for example, cases where a record referred to “copolyamide” while the corresponding context used the abbreviated form “COPA”. Such variations highlight the inherent challenges of entity normalization when text mining materials information. This balanced precision–recall profile is particularly suitable for constructing a training data set, as it provides both comprehensive coverage for effective model training and reliability to minimize the introduction of misleading examples.

**Table 3:** Performance of Property Extraction Evaluated against Manual Annotations (Precision, Recall, F1 Score in %)

property	precision	recall	F1
ultimate tensile strength	87.76	86.00	86.87
yield strength	89.47	94.44	91.82
fracture strength	75.00	75.00	75.00
Young’s modulus	62.50	83.33	71.43
ductility	72.22	92.86	81.25
all properties^a^	83.76	89.09	86.34

a All property values were computed by aggregating TP, FP, and FN across all properties, not by averaging the per-property scores.

In addition to the aggregated evaluation scoring metrics, the per-property evaluation revealed substantial variation in extraction accuracy across different mechanical properties (Table 3). Yield strength exhibited the highest overall performance, with precision of 89.47%, recall of 94.44%, and an F1 score of 91.82%, reflecting both the abundance of examples and the relatively consistent terminology used in the literature. This is likely because yield strength is reported in a highly consistent manner, often using standardized expressions such as yield stress or σ_*y*_. So, the applied pattern-matching rules and filters were highly effective, and the abundance of examples facilitated reliable recognition of this property. UTS also achieved high performance (F1 score = 86.87%), although with slightly lower recall. The slight drop in recall suggests a few UTS mentions might have been missed, possibly due to more varied terminology (e.g., “tensile strength at break” might not match our patterns).

For ductility, intermediate performance in information-extraction was achieved, with a recall of 92.86% but lower precision of 72.22%. Ductility is reported in diverse forms, including percent elongation and percent reduction in area, and the information-extraction workflow was able to capture most of these variations, yielding high recall. However, this flexibility came at the expense of precision, as some inaccuracies were introduced. A number of false positives appear to have arisen when percentages occurred near terms such as “elongation” but did not represent actual ductility measurements. Although exclusion rules were implemented to filter comparative statements, ductility descriptions remain difficult to parse reliably. For instance, “an elongation of 5% was observed” represents a valid measurement, whereas “elongation increased by 5%” does not.

The weakest performance was on Young’s modulus and fracture strength. Extraction of Young’s modulus achieved a high recall (approximately 83.33%) but only 62.50% precision, yielding an F1 score of 71.44%. The term, modulus, occurs in diverse contexts, including references to other moduli such as “bulk modulus” or as part of composite terms, which likely allowed some non-Young’s modulus values to be included. In addition, authors of scientific literature in this domain of research tend to employ varied terminology (e.g., elastic modulus, initial stiffness), making regular-expression-based matching difficult and occasionally overinclusive. Fracture strength achieved an F1 score of 75%, with both precision and recall at 75%. As the least represented property in the MechQA data set, with only a few hundred validation instances, the evaluation sample for fracture strength was limited. The modest evaluation scores likely reflect the challenge of capturing a relatively rare property, since the information-extraction patterns may not have covered all phrasings that authors in this field of research may use to describe fracture or failure strength.

The MechQA data set demonstrates high accuracy and coverage across the five target properties, with particularly strong performance for the two most frequently reported properties. The remaining errors are primarily attributable to the inherent complexity and variability of technical language in materials-science texts. The release of this data set provides a high-confidence training resource for the community, while recognizing that further extension to additional properties and refinement of extraction rules, especially for underrepresented terminology, could further enhance its quality.

### Model Development and Evaluation

#### Model Training and Optimization

The training dynamics of both the BERT-domain model (the BERT-base-cased model fine-tuned on the MechQA data set) and the LLaMA-domain model (the LLaMA-3.1–8B-Instruct model fine-tuned with LoRA on the MechQA data set) were monitored to ensure stable convergence and effective learning. As illustrated in Figure 6, training losses decreased asymptotically with an increasing number of training steps, demonstrating the expected rapid decline at the beginning of training followed by gradual stabilization. Although not a definitive measure of generalization, a decreasing loss indicates that the language models are learning useful representations from the QA data set. For both the BERT- and LLaMA-domain models, the loss curves declined over time and gradually flattened, suggesting that the selected training schedules were sufficient to achieve convergence.

**Figure 6:**
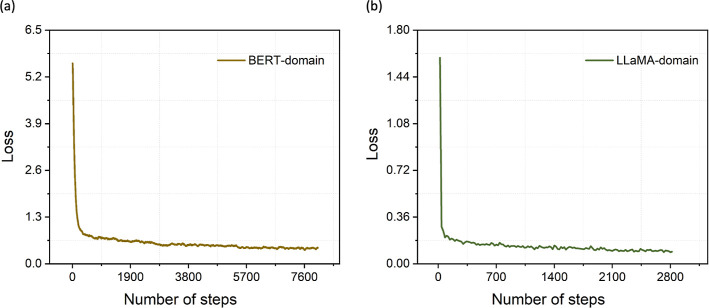
Training loss curves during the fine-tuning of LLMs obtained by exponential moving average: (a) BERT-domain model (over 3 epochs); (b) LLaMA-domain model (over 1 epoch).

The BERT-domain model exhibited a relatively high initial loss that decreased sharply during the first epoch and stabilized at approximately 0.4 by the third epoch, a pattern consistent with prior fine-tuning studies of BERT variants in scientific domains.^16,18^ In contrast, the LLaMA-domain model, fine-tuned with LoRA for a single epoch, began with a lower initial loss and followed a smooth downward trajectory, converging to approximately 0.1 at the end of training. This behavior is in line with observations from other LLaMA-based models that have been adapted to scientific tasks.^56,57^ Despite the shorter training horizon, the lack of divergence and continued reduction in loss suggest that the model successfully adapted to the task under the given resource constraints.

Training loss curves for XLNet, fine-tuned using the BERT-optimized hyperparameter configuration, are provided in the Supporting Information SII, which demonstrate stable convergence under this setting.

Hyperparameter optimization sweeps were conducted for both the BERT and LLaMA models to determine configurations that yielded the most effective fine-tuning performance. The results of these sweeps are illustrated in Figure 7, and more detailed results for each model are provided in the Supporting Information SIII.

**Figure 7:**
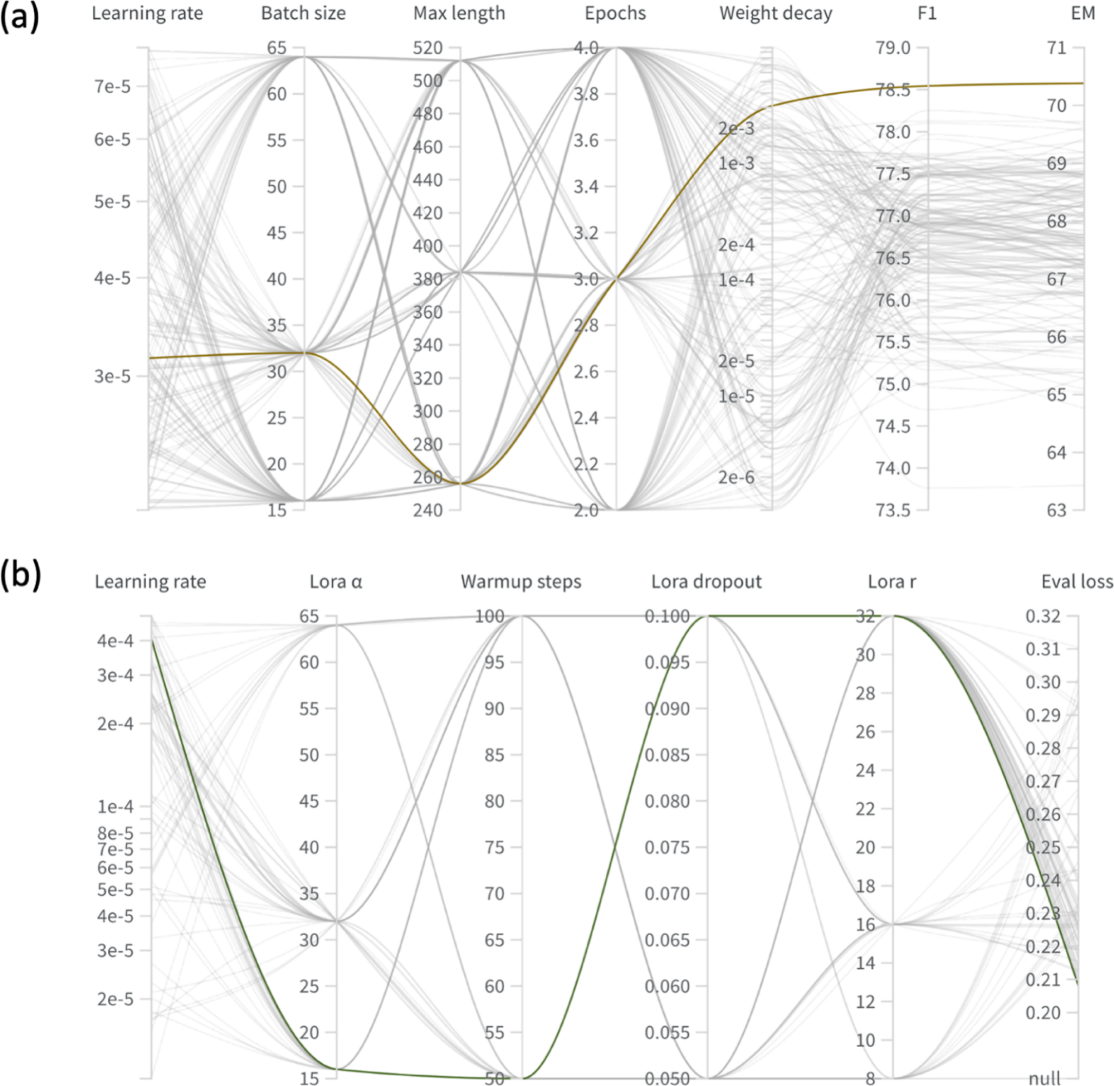
Hyperparameter sweep results for (a) the BERT-domain models and (b) the LLaMA-domain models. The optimal trial is highlighted for each language model by a bold line in each plot.

Figure 7a presents the sweep results for the BERT model. The optimal setting was obtained with a learning rate of 3.16 × 10^–5^, weight decay of 3.10 × 10^–3^, a batch size of 32, a maximum sequence length of 256, and three training epochs. Extending the maximum sequence length to 512 tokens did not provide any benefit, likely because most contexts were shorter than this threshold. The selected configuration (highlighted in bold) achieved the highest EM score on the validation subset.

For the LLaMA model, the Bayesian hyperparameter search identified the best-performing configuration with a learning rate of 4.046 × 10^–4^, a LoRA rank (*r*) of 32, a scaling factor (α) of 16, a dropout rate of 0.1, and 50 warmup steps (Figure 7b). The relatively high LoRA rank and scaling field indicate that a substantial level of low-rank adaptation was required for the model to effectively transfer its pretrained general linguistic knowledge to the specialized vocabulary and contextual patterns of materials science.

These optimal settings for both language models are summarized in Table 4 for clarity and reproducibility. These configurations are likely close to optimal for comparable tasks, such as domain-specific QA on scientific texts, and may therefore serve as a useful reference for future work using similar model architectures.

**Table 4:** Optimal Hyperparameters Identified for Each Fine-Tuned Model

model	hyperparameter	optimal value
BERT-base-cased	learning rate	3.16 × 10^–5^
	weight decay	3.10 × 10^–3^
	batch size	32
	max sequence length	256
	epochs	3
LLaMA-3.1–8B-instruct	learning rate	4.046 × 10^–4^
	LoRA rank (r)	32
	LoRA scaling factor (α)	16
	LoRA dropout	0.1
	warmup steps	50

#### Overall Performance Comparison

The performance of the three fine-tuned language models on the materials QA task was assessed and compared against the performance of relevant benchmarks. This evaluation demonstrates the effectiveness of the MechQA data set as a fine-tuning resource for domain-specific QA. Table 5 summarizes the performance of all evaluated language models on the MechQA validation set, with a visual comparison provided in Figure 8.

**Table 5:** Overall Performance of Evaluated Language Models on the MechQA Validation Set

model	EM (%)	F1 (%)	ECE(%)
BERT-SQuAD	34.40	50.25	27.09
BERT-domain	78.03	84.50	7.98
XLNet-SQuAD	35.27	52.22	25.76
XLNet-domain	78.21	84.70	6.25
LLaMA-3.1–8B-instruct	37.31	57.12	58.14
LLaMA-domain	80.48	86.25	8.08

**Figure 8:**
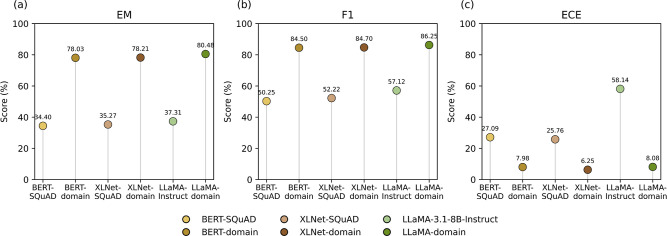
Performance comparison of evaluated language models on the MechQA validation set based on: (a) exact match; (b) F1 scores; (c) ECE.

The reported EM, F1, and ECE metrics quantify both answer accuracy and the reliability of model confidence estimates. Domain-specific fine-tuning resulted in substantial and consistent performance gains across all architectures. For extractive models, BERT and XLNet fine-tuned on MechQA demonstrated strong performance, achieving EM scores of 78.03% and 78.21%, respectively, along with corresponding F1 scores of 84.50% and 84.70%. In contrast, their benchmark counterparts (fine-tuned on SQuAD v1) afforded lower scores on the same questions, with EM scores near 35% and F1 scores around 50%. This corresponds to improvements of more than 40% in EM and over 30% in F1, highlighting that targeted fine-tuning on a domain-specific QA data set yields language models that are substantially more effective in the corresponding domain than models trained solely on generic English QA data. Domain-specific fine-tuning also substantially reduced calibration error, lowering ECE from approximately 26–27% to below 8%, indicating more reliable confidence estimates.

The LLaMA-domain model achieved the highest overall performance, with an EM score of 80.48% and an F1 score of 86.25%. This performance is very similar to the domain-specific fine-tuned extractive models, showing slight overall superiority by about 2% on both metrics. However, the zero-shot LLaMA-3.1–8B-Instruct model only achieved an EM score of 37.31% and an F1 score of 57.12% and exhibited severe miscalibration (ECE = 58.14%), despite the large parameter count and strong general-language capabilities. Even when prompted to “only output the answer,” the zero-shot model occasionally produced verbose responses, such as “The yield strength is 496 MPa” instead of the concise target answer “496 MPa,” indicating limited alignment with task-specific output conventions. After LoRA-based domain adaptation, the LLaMA model more than doubled its EM accuracy to approximately 80%, improved its F1 score by nearly 30%, and reduced ECE to approximately 8%, bringing its confidence reliability in line with that of the extractive models.

A comparison between the fine-tuned architectures shows that the 110M-parameter extractive models, after domain-specific fine-tuning, achieve performance that is essentially equivalent to that of the 8B-parameter LLaMA model on this task. The extractive models reached F1 scores of approximately 85%, while the generative model performed only slightly better. These findings underscore a resource-efficient path to strong performance, demonstrating that LLMs are not strictly required when high-quality domain-specific training data are available. Both domain-specific fine-tuned models outperformed general-purpose models such as GPT-4, achieving 62% accuracy on the MaScQA data set.^24^

Beyond differences in model scale and architecture, the evaluated approaches also differ substantially in computational cost during both training and inference. Fine-tuning the extractive SLMs required less than 1 h on a single node with four NVIDIA A100 GPUs, whereas adapting the LLaMA-based models using LoRA required approximately three to 4 h on the same hardware. Inference-time efficiency exhibited an even larger disparity: including ECE computation, inference for the domain-specific LLaMA model required approximately 4 h, and the zero-shot LLaMA model required approximately 6 h, while all extractive models completed inference within approximately 10 min. This contrast highlights the substantially higher practical cost of generative models, even when PEFT techniques are employed.

For safety-critical materials applications, these results also have implications for uncertainty handling and deployment. The substantially lower ECE values of the domain-specific models (below 8%) compared to general-purpose benchmarks (26–58%) indicate that their confidence scores can reliably be used to flag low-confidence predictions for downstream filtering or human review. Architectural differences also influence deployment considerations: extractive models constrain outputs to spans of the source text, ensuring provenance and verifiability, whereas generative models provide greater output flexibility but require careful prompt engineering to maintain consistency of output format. Safe deployment in materials applications benefits from explicit confidence thresholds and human-in-the-loop verification, particularly for low-confidence predictions. In addition, the integration of domain-specific QA models as specialized tools within larger agentic or multiagent frameworks enables higher-level components to manage uncertainty assessment and determine when human expert input is required.

#### Performance by Question Type

Analysis of the performance of the language models by question type provides additional insights. Model performance stratified by question type is reported in Table 6 and illustrated in Figure 9.

**Table 6:** Per-question-type Performance of Evaluated Language Models on the MechQA Validation Set

models	entity lookup			numeric lookup			property identification		
	EM	F1	ECE	EM	F1	ECE	EM	F1	ECE
BERT-SQuAD	35.18	43.83	26.91	36.99	55.56	29.01	31.02	51.37	25.36
BERT-domain	75.60	79.06	6.77	75.13	85.38	7.93	83.37	89.07	10.81
XLNet-SQuAD	26.91	39.54	29.25	38.04	57.53	27.54	40.86	59.59	20.49
XLNet-domain	75.67	79.45	7.97	75.04	85.31	6.33	83.93	89.33	8.21
LLaMA-3.1–8B-instruct	25.48	44.02	71.90	46.18	62.25	51.26	40.28	65.09	51.59
LLaMA-domain	77.90	81.60	14.98	73.87	84.67	5.12	89.68	92.48	4.46

**Figure 9:**
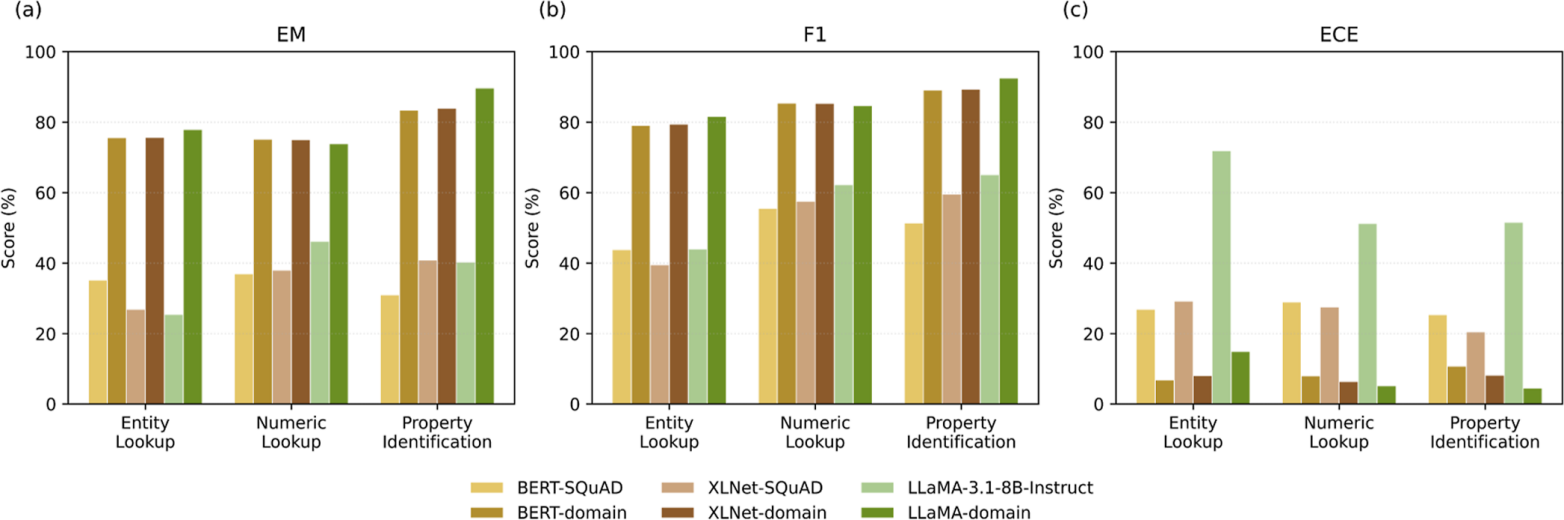
Per-question-type performance comparison of evaluated language models on the MechQA validation set based on: (a) exact match; (b) F1 scores; (c) ECE.

Across all domain-specific models, Property Identification questions consistently yielded the highest EM and F1 scores. These questions required identification of the correct property given a numerical value and material context. Consequently, both extractive and generative models were able to reliably associate numerical values with property labels. The slightly higher EM score of the LLaMA-domain-specific model reflects its stronger ability to reproduce the exact property names across varied phrasings, whereas the extractive domain-specific model, though comparably strong, occasionally returned semantically related or partially overlapping terms. Among these, the LLaMA-domain model achieved the lowest ECE, indicating both strong accuracy and well-calibrated confidence when reproducing exact property names.

Numeric Lookup questions highlighted a structural advantage of extractive QA formulations. Extractive models such as BERT-domain and XLNet-domain returned exact numeric spans from the context, preserving both values and units (e.g., “350 MPa”), which translated into higher EM scores under strict evaluation criteria. In contrast, the LLaMA-domain-specific model occasionally reformatted answers, such as omitting the unit (“350”) or appending unnecessary tokens (“at 350 MPa” with an “at”). These formatting variations negatively affected its EM score, even when the semantic content was correct. This highlights a known limitation of generative models: maintaining strict adherence to expected output formats, particularly for values with units.

Entity Lookup questions posed the greatest challenge across architectures and exhibited the highest calibration error. This task requires identifying the correct material name associated with a reported property value, where entity surface forms may vary substantially due to abbreviations, stoichiometric notation, or trade names. Extractive models are sensitive to span-boundary ambiguity, while generative models must implicitly normalize entities under uncertainty. Although the LLaMA-domain model achieved marginally higher F1 scores, the elevated ECE observed for this category indicates that Entity Lookup primarily tests normalization robustness rather than simple extraction accuracy.

Across question types, these results illustrate how the architectural design of a language model shapes its behavior across different question types. The extractive domain-specific models demonstrate strong precision in retrieving numeric and property values directly from text, while the generative domain-specific model shows greater understanding in handling linguistic variation and normalization. More detailed validation assessment is given in the Supporting Information SIV.

#### Performance by Mechanical Property

Table 7 summarizes the QA performance of the domain-specific models stratified by mechanical property and is illustrated in Figure 10. This analysis examines ultimate tensile strength, yield strength, fracture strength, Young modulus, and ductility to provide a property-specific comparison of evaluated language models on the MechQA validation set.

**Table 7:** Per-Mechanical-Property Performance of Evaluated Language Models on the MechQA Validation Set

model	UTS			yield strength			fracture strength			Young’s modulus			ductility		
	EM	F1	ECE	EM	F1	ECE	EM	F1	ECE	EM	F1	ECE	EM	F1	ECE
BERT-SQuAD	35.80	52.85	26.00	35.10	52.01	26.77	42.44	61.31	20.71	23.69	36.85	36.37	34.74	44.76	25.37
BERT-domain	77.37	84.30	8.44	76.44	84.29	7.75	86.05	90.21	10.15	76.76	83.62	7.23	84.57	85.38	8.01
XLNet-SQuAD	37.94	55.48	23.40	34.78	53.43	26.57	44.77	63.84	18.86	24.23	38.62	35.81	34.70	46.05	24.53
XLNet-domain	77.79	84.68	6.67	76.74	84.46	6.54	85.08	89.78	8.08	75.87	82.90	6.78	84.70	85.91	4.09
LLaMA-3.1-8B-instruct	38.83	58.70	56.64	36.98	59.94	58.98	50.39	70.61	44.95	36.70	54.54	61.23	30.56	42.15	64.92
LLaMA-domain	79.82	85.82	10.14	78.68	86.09	9.67	88.95	91.92	5.78	81.45	86.74	7.75	85.64	86.61	9.77

**Figure 10:**
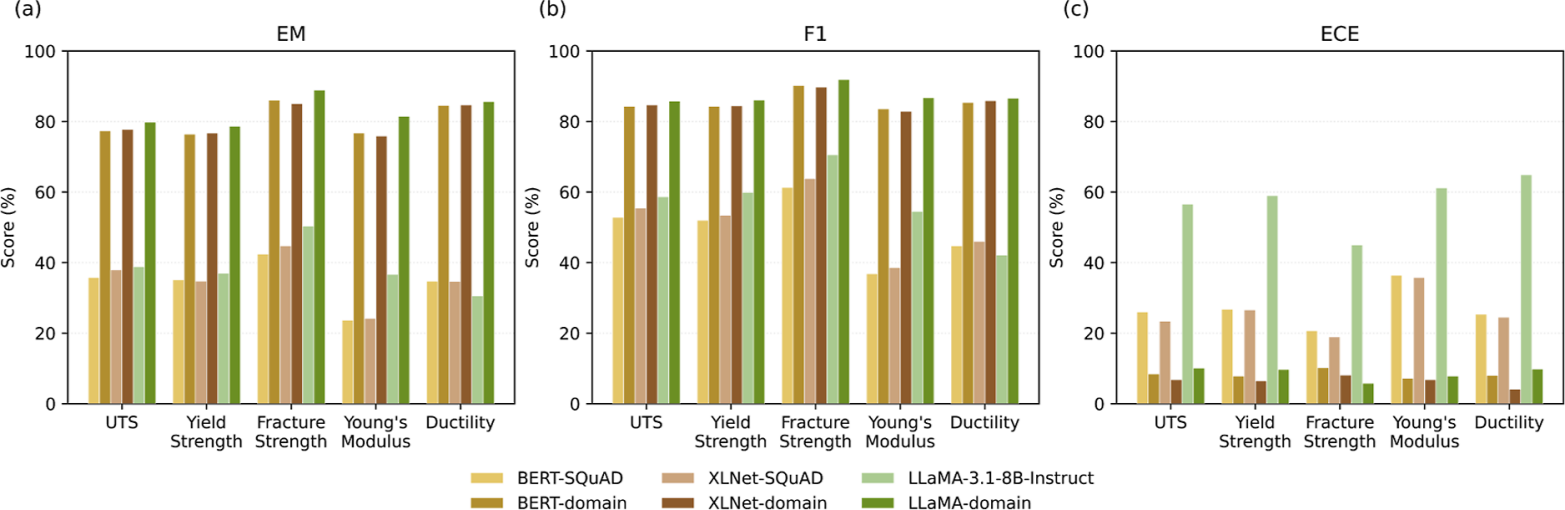
Per-mechanical-property performance comparison of evaluated language models on the MechQA validation set based on: (a) exact match; (b) F1 scores; (c) ECE.

Yield strength and UTS consistently achieved the strongest QA performance across all domain-specific models, with EM scores exceeding approximately 76% and F1 scores above 84%. These properties were also associated with the highest precision and recall during data set construction, reflecting both consistent terminology and abundant representation in the source literature. The alignment between data set extraction quality and downstream QA performance suggests that cleaner supervision signals facilitate not only higher accuracy but also improved calibration.

In contrast, Young’s modulus and fracture strength were systematically more challenging. Young’s modulus is linguistically ambiguous, with multiple synonyms and contextual uses of the term “modulus,” which likely introduced residual noise during data set construction. Fracture strength, as a less frequently reported property, exhibited greater variability in phrasing and lower statistical support. These factors manifested as lower EM scores and higher calibration error across models, indicating that QA performance is constrained by linguistic variability and data sparsity rather than model capacity alone.

Ductility exhibited intermediate performance, reflecting its heterogeneous linguistic expression in the literature, including percentage elongation and related strain-based descriptors. While domain adaptation substantially improved both accuracy and calibration for this property, variability in phrasing continued to pose challenges relative to strength-based properties.

Across mechanical properties, extractive and generative models exhibited consistent relative performance trends, despite architectural differences. Both model families performed strongest on yield strength and UTS, and weakest on Young’s modulus and fracture strength. This convergence suggests that property-specific QA difficulty is driven primarily by linguistic variability and data availability rather than by model architecture. While extractive models maintained lower calibration error overall, and generative models achieved slightly higher peak accuracy on well-defined properties, neither architecture was able to fully overcome ambiguity and sparsity, where it was inherent to certain property categories. More detailed validation assessment is given in the Supporting Information SIV.

## Conclusions

This study has presented a knowledge-distillation method that autogenerates a large, domain-specific QA data set for materials engineering. The strategy behind this method involves reverse-engineering a large, structured, experimental materials data set on mechanical properties that had been mined from the academic literature using the materials-aware NLP toolkit, ChemDataExtractor. The MechQA data set, extracted from 125,967 materials-science articles, comprises 202,068 data records containing extracted contexts and QA pairs for the five fundamental mechanical properties of materials. Manual evaluation confirmed the high quality of the data set, with a precision of 83.76%, a recall of 89.09%, and an F1 score of 86.34%. This MechQA data set enables the domain-specific fine-tuning of both two extractive SLMs (the BERT-domain-specific model and the XLNet-domain-specific model) and a generative LLM (the LLaMA-domain-specific model), achieving high performance without the need for full model pretraining.

Fine-tuning a vanilla BERT-base-cased model on the MechQA data set yielded an F1 score of 84.50% and EM accuracy of 78.03%. This BERT-domain-specific performance is substantially better than that of its BERT-SQuAD benchmark and affords, to our knowledge, a new state-of-the-art performance for materials-property QA tasks using BERT models. A similarly strong performance was observed for the XLNet-domain model, demonstrating that the benefits of domain-specific supervision generalize across extractive transformer architectures. This result highlights the effectiveness of specialized models: when paired with relevant data, even a relatively small transformer can reach expert-level accuracy. The LLaMA-3.1–8B-Instruct model, fine-tuned with LoRA, achieved comparable performance (86.25% F1), confirming that large instruction-tuned models can adapt to precise numeric extraction tasks. However, qualitative analysis revealed that the LLaMA model occasionally produced minor errors in phrasing or formatting, which may be problematic for a strict database population, despite achieving strong accuracy and improved calibration after domain adaptation.

Our approach did not require training language models from scratch, nor did it rely on pretraining that necessitates excessive computational resources. By employing knowledge distillation in data form, the need to pretrain a new language model for materials science was eliminated. Fine-tuning of a vanilla BERT model required less than 1 h of computation on a single node with 4 NVIDIA A100 GPUs, while the comparable fine-tuning of the LLaMA-3.1 model with LoRA took a few hours, indicating that the extraction method is computationally practical for the academic research community. The integration of LoRA and quantization illustrates that LLMs can be adapted to commodity hardware with minimal loss in performance. This significantly reduces the barrier to developing domain-specific AI tools, as a researcher with access to a well-structured domain-specific data set can obtain a high-quality language model without requiring an industrial-scale computational budget, while maintaining reliable calibration across question types and property categories.

Future research could advance along several promising directions. The MechQA data set may be expanded to include additional mechanical or physical properties such as hardness, fatigue limit, or thermal characteristics, as well as more diverse classes of materials, thereby improving coverage. The exploration of few-shot learning and adapter fusion techniques could enable a single LLM to address multiple domains by incorporating different LoRA modules for distinct tasks. Recent advances in materials-focused AI have introduced complementary paradigms beyond text-centric models, including graph-based and graph-native reasoning systems,^58,59^ agentic and swarm-based frameworks such as ProtAgents^60^ and Sparks,^61^ and emerging reasoning-centric architectures.^62–65^ Together, these approaches emphasize structured reasoning, hypothesis generation, knowledge graph construction, and autonomous scientific discovery, positioning them as synergistic with the QA-driven framework developed in this work.

Integration of these QA-enabled LLMs into user-facing platforms such as literature-search engines that are capable of answering domain-specific questions, or digital-laboratory assistants that summarize property data for specified materials, represents a critical step toward real-world applications. Furthermore, the strategy of automatic QA data set generation demonstrated in this work can be generalized to other scientific domains with rich literature, providing a pathway for broad adoption. The combination of automated knowledge distillation and fine-tuning has strong potential to become a standard workflow for the development of specialized LLMs across disciplines, thereby accelerating scientific discovery through improved access to knowledge embedded in publications.

In broad terms, the findings of this work demonstrate that with appropriate QA data sets, language models of modest size, i.e., SLMs, can achieve expert-level performance in specialized QA tasks. This provides a transferable framework for advancing applications in materials science and related domains, including off-line SLM utility, which can be critical for security applications, use in remote field work, or in the deployment of autonomous AI agents. This approach is expected to stimulate further research at the intersection of NLP and materials science, thereby contributing to more rapid and efficient innovation in the development of new materials.

## Supplementary Material



## Data Availability

The original materials data set containing the materials names and cognate values of the five fundamental mechanical properties is publicly available online at 10.6084/m9.figshare.25881025. The MechQA data set introduced in this work, including automatically generated QA pairs in SQuAD format, is available at: https://huggingface.co/datasets/mz516/MechQA-dataset. The three domain-specific fine-tuned language models developed in this study are also hosted on Hugging Face: https://huggingface.co/CambridgeMolecularEngineering. All scripts used to construct the MechQA data set from the original source, as well as the scripts used for model fine-tuning and evaluation, are available in the GitHub repository: https://github.com/mz-516/MechQA.

## Notes

CSV file containing the complete list of excluded compound names with corresponding removal criteria; XLNet training convergence analyses; detailed results of hyperparameter optimization sweeps for both BERT and LLaMA models, including parameter ranges explored and performance outcomes; and detailed performance evaluations stratified by mechanical property and question type (PDF)

## References

[1] Nabavi S. F., Garmestani H., Fekri F. (2025). AI-powered language models for alloy
design and laser-based manufacturing: A review of NLP applications
in materials science. J. Manuf. Process..

[2] Schmidt J., Marques M. R., Botti S., Marques M. A. (2019). Recent advances
and applications of machine learning in solid-state materials science. npj Comput. Mater..

[3] Himanen L., Geurts A., Foster A. S., Rinke P. (2019). Data-driven materials
science: status, challenges, and perspectives. Adv. Sci..

[4] Jiang X., Wang W., Tian S., Wang H., Lookman T., Su Y. (2025). Applications of natural language
processing and large language models
in materials discovery. npj Comput. Mater..

[5] Hu Y., Buehler M. J. (2023). Deep language models for interpretative and predictive
materials science. APL Mach. Learn..

[6] Seow W. L., Chaturvedi I., Hogarth A., Mao R., Cambria E. (2025). A review of
named entity recognition: from learning methods to modelling paradigms
and tasks. Artif. Intell. Rev..

[7] Sierepeklis O., Cole J. M. (2025). Autogenerating a domain-specific
question-answering
data aet from a thermoelectric materials database to enable high-performing
BERT models. JCIM.

[8] Choi J., Lee B. (2024). Accelerating materials language processing
with large language models. Commun. Mater..

[9] Sipilä M., Mehryary F., Pyysalo S., Ginter F., Todorović M. (2025). Question Answering
models for information extraction from perovskite materials science
literature. Commun. Mater..

[10] Cole J. M. (2020). A design-to-device
pipeline for data-driven materials discovery. Acc. Chem. Res..

[11] Dagdelen J., Dunn A., Lee S., Walker N., Rosen A. S., Ceder G., Persson K. A., Jain A. (2024). Structured information
extraction from scientific text with large language models. Nat. Commun..

[12] Miret S., Krishnan N. A., Sanchez-Lengeling B., Skreta M., Venugopal V., Wei J. N. (2024). Perspective on AI
for accelerated materials design
at the AI4MAT-2023 workshop at NeurIPS 2023. Digital Discovery.

[13] Lu W., Luu R. K., Buehler M. J. (2025). Fine-tuning
large language models
for domain adaptation: Exploration of training strategies, scaling,
model merging and synergistic capabilities. npj Comput. Mater..

[14] Beltagy I., Lo K., Cohan A. (2019). SciBERT: A
pretrained language model for scientific
text. arXiv preprint.

[15] Gupta T., Zaki M., Krishnan N. M. A., Mausam (2022). MatSciBERT:
A materials domain language
model for text mining and information extraction. npj Comput. Mater..

[16] Huang S., Cole J. M. (2022). BatteryBERT: A pretrained
language model for battery
database enhancement. JCIM.

[17] Zhao J., Huang S., Cole J. M. (2023). OpticalBERT and
OpticalTable-SQA:
Text-and table-based language models for the optical-materials domain. JCIM.

[18] Kumar P., Kabra S., Cole J. M. (2025). MechBERT:
Language models for extracting
chemical and property relationships about mechanical stress and strain. JCIM.

[19] Hurst A., Lerer A., Goucher A. P., Perelman A., Ramesh A., Clark A., Ostrow A., Welihinda A., Hayes A., Radford A. (2024). Gpt-4o
system card. arXiv preprint.

[20] Maslej N., Fattorini L., Perrault R., Gil Y., Parli V., Kariuki N., Capstick E., Reuel A., Brynjolfsson E., Etchemendy J. (2025). Artificial intelligence index report 2025. arXiv preprint.

[21] Cheung J. J., Shen S., Zhuang Y., Li Y., Ramprasad R., Zhang C. (2025). MSQA: Benchmarking LLMs on graduate-level
materials science reasoning
and knowledge. arXiv preprint.

[22] Fernandes A. A., Koehler M., Konstantinou N., Pankin P., Paton N. W., Sakellariou R. (2023). Data preparation: A technological perspective and review. SN Comput. Sci..

[23] Vidgen B., Derczynski L. (2020). Directions
in abusive language training data, a systematic
review: Garbage in, garbage out. PLoS One.

[24] Zaki M., Krishnan N. A. (2024). MaScQA:
investigating materials science knowledge
of large language models. Digital Discovery.

[25] Rajpurkar P., Zhang J., Lopyrev K., Liang P. (2016). SQuAD: 100,000+ questions
for machine comprehension of text. arXiv preprint.

[26] Li Z., Cole J. M. (2025). Auto-generating question-answering datasets with domain-specific
knowledge for language models in scientific tasks. Digital Discovery.

[27] Swain M. C., Cole J. M. (2016). ChemDataExtractor:
a toolkit for automated extraction
of chemical information from the scientific literature. JCIM.

[28] Mavracic J., Court C. J., Isazawa T., Elliott S. R., Cole J. M. (2021). ChemDataExtractor
2.0: Autopopulated ontologies for materials science. JCIM.

[29] Isazawa T., Cole J. M. (2022). Single model for
organic and inorganic chemical named
entity recognition in ChemDataExtractor. JCIM.

[30] Kumar P., Kabra S., Cole J. M. (2024). A Database of stress-Strain
properties
auto-generated from the scientific literature using ChemDataExtractor. Sci. Data.

[31] McKinney W. (2010). Data structures for
statistical computing in Python. Scipy.

[32] Devlin, J. ; Chang, M.-W. ; Lee, K. ; Toutanova, K. Bert: Pre-training of deep bidirectional transformers for language understanding. In Proceedings of the 2019 conference of the North American Chapter of the association for computational linguistics: human language technologies, volume 1 (long and short papers); Association for Computational Linguistics, 2019, pp 4171–4186.

[33] Yang, Z. ; Dai, Z. ; Yang, Y. ; Carbonell, J. ; Salakhutdinov, R. R. ; Le, Q. V. Xlnet: Generalized autoregressive pretraining for language understanding. In Proceedings of the 33rd International Conference on Neural Information Processing Systems; ACM, 2019, pp 5753–5763.

[34] Grattafiori A., Dubey A., Jauhri A., Pandey A., Kadian A., Al-Dahle A., Letman A., Mathur A., Schelten A., Vaughan A. (2024). The LLaMA 3 herd of
models. arXiv
preprint.

[35] Pyzer-Knapp E. O., Manica M., Staar P., Morin L., Ruch P., Laino T., Smith J. R., Curioni A. (2025). Foundation
models for
materials discovery–current state and future directions. npj Comput. Mater..

[36] Liu Y., Ott M., Goyal N., Du J., Joshi M., Chen D., Levy O., Lewis M., Zettlemoyer L., Stoyanov V. (2019). RoBERTa: A robustly optimized BERT
pretraining approach. arXiv preprint.

[37] He P., Liu X., Gao J., Chen W. (2020). DeBERTa: Decoding-enhanced
BERT with
disentangled attention. arXiv preprint.

[38] Kumar S., Singh S. (2024). Fine-tuning LLaMA 3 for sentiment
analysis: Leveraging AWS cloud
for enhanced performance. SN Comput. Sci..

[39] Workshop B., Scao T. L., Fan A., Akiki C., Pavlick E., Ilić S., Hesslow D., Castagné R., Luccioni A. S., Yvon F. (2022). Bloom: A 176b-parameter
open-access multilingual language model. arXiv
preprint.

[40] Black S., Biderman S., Hallahan E., Anthony Q., Gao L., Golding L., He H., Leahy C., McDonell K., Phang J. (2022). GPT-NEOx-20B: An open-source autoregressive language
model. arXiv preprint.

[41] Borzunov A., Baranchuk D., Dettmers T., Ryabinin M., Belkada Y., Chumachenko A., Samygin P., Raffel C. (2022). Petals: Collaborative
inference and fine-tuning of large models. arXiv
preprint.

[42] Arabadzhieva-Kalcheva, N. ; Kovachev, I. Comparison of BERT and XLNet accuracy with classical methods and algorithms in text classification. In 2021 International Conference on Biomedical Innovations and Applications (BIA); IEEE, 2022, pp 74–76.

[43] Wu Y., Schuster M., Chen Z., Le Q. V., Norouzi M., Macherey W., Krikun M., Cao Y., Gao Q., Macherey K. (2016). Google’s neural machine translation
system: Bridging the gap between human and machine translation. arXiv preprint.

[44] Kudo T., Richardson J. (2018). SentencePiece:
A simple and language independent subword
tokenizer and detokenizer for neural text processing. arXiv preprint.

[45] Hu, E. J. ; Shen, Y. ; Wallis, P. ; Allen-Zhu, Z. ; Li, Y. ; Wang, S. ; Wang, L. ; Chen, W. ; LoRA: Low-rank adaptation of large language models. In International Conference on Learning Representations; ICLR, 2022, p 3.

[46] Dettmers, T. ; Zettlemoyer, L. The case for 4-bit precision: k-bit inference scaling laws. In ICML’23: Proceedings of the 40th International Conference on Machine Learning; International Conference on Machine Learning, 2023, pp 7750–7774.

[47] Rasley, J. ; Rajbhandari, S. ; Ruwase, O. ; He, Y. Deepspeed: System optimizations enable training deep learning models with over 100 billion parameters. In Proceedings of the 26th ACM SIGKDD international conference on knowledge discovery & data mining; ACM, 2020, pp 3505–3506.

[48] Biewald, L. ; Experiment Tracking with Weights and Biases, 2020.

[49] Joshi M., Chen D., Liu Y., Weld D. S., Zettlemoyer L., Levy O. (2020). SpanBERT: Improving pre-training
by representing and predicting spans. Trans.
Assoc. Comput. Linguist..

[50] Chen, A. ; Stanovsky, G. ; Singh, S. ; Gardner, M. Evaluating question answering evaluation. In Proceedings of the 2nd workshop on machine reading for question answering; Association for Computational Linguistics, 2019, pp 119–124.

[51] Naeini, M. P. ; Cooper, G. ; Hauskrecht, M. Obtaining well calibrated probabilities using bayesian binning. In Proceedings of the AAAI conference on artificial intelligence; ACM, 2015, pp 2901–2907. PMC4410090 25927013

[52] Guo, C. ; Pleiss, G. ; Sun, Y. ; Weinberger, K. Q. On calibration of modern neural networks. In Proceedings of the 34 th International Conference on Machine; International Conference on Machine Learning, 2017, pp 1321–1330.

[53] Si C., Zhao C., Min S., Boyd-Graber J. (2022). Re-examining
calibration: The case of question answering. arXiv preprint.

[54] Lowerre, B. T. The Harpy Speech Recognition System; Carnegie Mellon University, 1976.

[55] Yang Y., Dan S., Roth D., Lee I. (2023). On the calibration of multilingual
question answering llms. arXiv preprint.

[56] Gruver N., Sriram A., Madotto A., Wilson A. G., Zitnick C. L., Ulissi Z. (2024). Fine-tuned language models generate
stable inorganic
materials as text. arXiv preprint.

[57] Mishra V., Singh S., Ahlawat D., Zaki M., Bihani V., Grover H. S., Mishra B., Miret S., Krishnan N. (2024). Foundational large language models for materials research. arXiv preprint.

[58] Buehler M. J. (2025). In Situ
Graph Reasoning and Knowledge Expansion Using Graph-PRefLexOR. Adv. Intell. Syst..

[59] Buehler M. J. (2024). Accelerating
scientific discovery with generative knowledge extraction, graph-based
representation, and multimodal intelligent graph reasoning. Mach. Learn.: Sci. Technol..

[60] Ghafarollahi A., Buehler M. J. (2024). ProtAgents: protein
discovery via large language model
multi-agent collaborations combining physics and machine learning. Digital Discovery.

[61] Ghafarollahi A., Buehler M. J. (2025). Sparks:
Multi-agent artificial intelligence model discovers
protein design principles. arXiv preprint.

[62] Ghafarollahi A., Buehler M. J. (2025). SciAgents: automating scientific
discovery through
bioinspired multi-agent intelligent graph reasoning. Adv. Mater..

[63] Jaech A., Kalai A., Lerer A., Richardson A., El-Kishky A., Low A., Helyar A., Madry A., Beutel A., Carney A. (2024). Openai
o1 system card. arXiv preprint.

[64] Zhang K., Zuo Y., He B., Sun Y., Liu R., Jiang C., Fan Y., Tian K., Jia G., Li P. (2025). A survey
of reinforcement learning for large reasoning models. arXiv preprint.

[65] Buehler M. J. (2025). Preflexor:
Preference-based recursive language modeling for exploratory optimization
of reasoning and agentic thinking. npj Artif.
Intell..

