# Supplementary material for: Automatic Generation of a Mechanical Properties Question-Answering Data Set for Language Model Benchmarking: A Comparative Study of BERT, XLNet, and LLaMA Models
Source: J Chem Inf Model. 2026 Mar 27;66(7):3840–57. doi: 10.1021/acs.jcim.5c02646 (PMC13080984; doi:10.1021/acs.jcim.5c02646)
Supplement: Supplementary file 1 [file ci5c02646_si_001.pdf]

Supporting Information: Automatic Generation of a Mechanical Properties Question-Answering Dataset for Language Model Benchmarking: A Comparative Study of BERT, XLNet, and LLaMA Models

Minglei Zhang and Jacqueline M. Cole*

Ray Dolby Centre, Cavendish Laboratory, Department of Physics, University of Cambridge, J. J. Thomson Avenue, Cambridge CB3 0US. U.K.

E-mail: jmc61@cam.ac.uk

SI. Compound Inclusion and Exclusion Criteria

From the original dataset, 4,793 spurious or ambiguous compound entries were removed based on predefined criteria (e.g., non-standard naming conventions, duplicate aliases, missing or inconsistent property values, and unresolved structural ambiguities). Due to the large number of entries, the full exclusion list is provided separately in the file removed_compounds.csv. For illustration, a representative subset of excluded compounds and their removal criteria is shown in Table S1.

Table S1: Representative excluded compounds and their removal criteria.[a]

Compound	Count	Removal criterion
steel	27,777	generic term
concrete	8,022	generic term
Al	5,582	generic term
aluminum	5,569	generic term
carbon	5,266	frequent & non-specific
steels	5,197	generic term
Cu	3,660	generic term
epoxy	3,177	frequent & non-specific
...		

[a] Only a representative subset is shown here; the full exclusion list of 4,793 compounds with removal criteria is available in removed_compounds.csv.

SII. XLNet Training Performance

Training loss curves for XLNet, fine-tuned using the same BERT-optimized hyperparameter configuration, are provided in Figure S1. These results demonstrate that the XLNet model converges smoothly and remains numerically stable throughout training. The loss decreases rapidly during the initial training steps and continues with a gradual monotonic decline, consistent with effective optimization under this configuration. Together with the BERT-domain and LLaMA-domain models shown in the main manuscript, these results confirm that the selected hyperparameters generalize well across model architectures and support reliable convergence during domain adaptation.

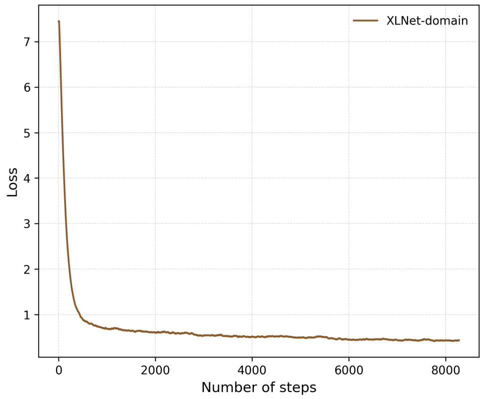

Figure S1: Training loss curves during the fine-tuning of XLNet obtained by exponential moving average (fine-tuned under the BERT-optimized hyperparameter configuration).

SIII. Hyperparameter Optimization

Comprehensive hyperparameter optimization sweeps were first carried out for the extractive BERT model, where multiple values of learning rate, batch size, dropout probability, sequence length, and number of training epochs were systematically explored. Performance outcomes were evaluated using validation accuracy, F1-score, and loss trends, and the full results are presented in Table S2.

Table S2: Comprehensive hyperparameter sweep results for the BERT model.

Name	learning_rate	batch_size	max_length	epochs	weight_decay	f1	exact_match
celestial-sweep-59	3.162e-05	32	256	3	0.003103	78.54	70.38
vocal-sweep-33	5.134e-05	32	256	3	0.001539	78.13	69.71
vocal-sweep-137	3.88e-05	32	256	3	8.457e-05	78.25	69.67
stilted-sweep-1	3.219e-05	16	256	3	0.0006482	77.68	69.62
wobbly-sweep-6	2.925e-05	16	384	4	0.0007559	78.1	69.57
clear-sweep-84	3.483e-05	32	256	3	0.00478	77.47	69.43
glad-sweep-93	3.56e-05	32	384	4	0.001375	77.74	69.38
warm-sweep-80	4.524e-05	16	256	2	4.112e-06	77.97	69.19
helpful-sweep-55	3.054e-05	16	256	4	0.000303	77.6	69.19
splendid-sweep-67	2.851e-05	16	256	4	0.0005513	77.48	69.1
swept-sweep-127	4.356e-05	64	256	3	1.607e-06	77.52	69.1
brisk-sweep-12	2.451e-05	16	256	3	0.0008052	77.68	69.05
playful-sweep-119	2.23e-05	16	256	4	2.543e-05	77.38	69
playful-sweep-3	5.785e-05	32	512	4	0.0007585	77.72	69
absurd-sweep-113	5.532e-05	32	256	4	0.0004049	77.48	69
rural-sweep-39	2.429e-05	16	256	4	3.122e-06	77.28	68.9
smart-sweep-18	2.46e-05	16	256	3	0.006931	77.45	68.86
amber-sweep-13	2.447e-05	32	256	4	9.764e-06	77.47	68.86
peach-sweep-90	3.078e-05	16	256	4	0.002002	77.51	68.86
whole-sweep-149	3.53e-05	32	384	4	0.001273	77.68	68.81
grateful-sweep-150	7.836e-05	32	256	4	2.876e-06	77.22	68.81
fallen-sweep-74	4.178e-05	32	256	2	7.983e-05	77.58	68.81
faithful-sweep-145	6.252e-05	16	256	3	0.002023	76.88	68.81
giddy-sweep-57	2.369e-05	32	256	3	5.598e-06	77.53	68.81
smart-sweep-49	4.552e-05	32	256	4	0.0005837	77.25	68.81
stellar-sweep-148	4.663e-05	16	512	4	0.0002122	77.61	68.81
effortless-sweep-25	3.673e-05	16	256	2	0.0004228	77.57	68.76

Name	learning_rate	batch_size	max_length	epochs	weight_decay	f1	exact_match
robust-sweep-146	3.015e-05	16	512	4	8.662e-06	77.49	68.76
stoic-sweep-82	4.111e-05	16	512	4	0.0003107	77.91	68.71
desert-sweep-15	3.427e-05	32	256	4	0.0005029	77.05	68.67
zany-sweep-125	5.675e-05	32	512	4	0.0009015	77.02	68.67
misunderstood-sweep-98	2.926e-05	32	256	3	5.299e-06	77.16	68.62
silver-sweep-76	4.567e-05	16	384	3	0.001996	77.46	68.57
ethereal-sweep-79	2.64e-05	32	384	4	0.0001484	77.59	68.57
unique-sweep-71	7.284e-05	32	256	4	0.001675	77.25	68.57
ruby-sweep-31	2.677e-05	16	256	4	1.652e-06	77.02	68.52
fancy-sweep-88	2.762e-05	16	384	4	0.002383	77.07	68.52
easy-sweep-139	4.398e-05	64	256	4	0.000572	77.3	68.52
proud-sweep-120	5.155e-05	64	256	4	1.039e-06	76.77	68.48
comfy-sweep-89	3.509e-05	32	256	4	0.0008659	76.98	68.48
feasible-sweep-152	2.423e-05	16	384	4	5.056e-05	77.49	68.48
still-sweep-65	2.311e-05	16	256	3	1.837e-05	77.5	68.48
rich-sweep-64	5.545e-05	32	384	4	3.961e-06	77.25	68.43
woven-sweep-115	3.867e-05	16	384	4	0.001039	77.55	68.43
dauntless-sweep-70	7.57e-05	64	256	4	0.007571	77.04	68.43
polar-sweep-133	3.493e-05	32	384	4	0.002691	77.42	68.38
graceful-sweep-158	2.448e-05	32	384	4	0.00675	77.6	68.33
azure-sweep-100	3.397e-05	32	384	3	0.004439	76.91	68.33
sweet-sweep-37	5.575e-05	16	256	2	0.002379	76.93	68.33
revived-sweep-132	4.057e-05	64	256	3	0.001726	76.89	68.29
earthy-sweep-153	7.008e-05	32	384	4	0.001319	76.81	68.29
sweet-sweep-4	5.061e-05	32	512	4	0.0007814	76.87	68.29
usual-sweep-135	3.858e-05	32	384	3	0.0001339	77.49	68.29
glad-sweep-129	2.39e-05	64	256	4	1.23e-06	77.19	68.24
bright-sweep-111	4.497e-05	64	256	2	1.115e-06	77.2	68.24
vital-sweep-47	7.77e-05	64	512	4	0.0002271	77.18	68.24
silver-sweep-105	2.733e-05	16	384	4	2.147e-05	77.25	68.14
northern-sweep-43	3.163e-05	32	512	4	1.127e-05	76.91	68.14
faithful-sweep-155	6.003e-05	16	256	3	0.0004715	76.77	68.14
drawn-sweep-101	5.778e-05	16	512	3	0.0001299	76.95	68.1
neat-sweep-92	3.067e-05	32	384	4	0.002126	77.19	68.1
dashing-sweep-9	3.048e-05	16	384	4	0.001394	77.69	68.1
stilted-sweep-140	3.826e-05	32	256	4	0.006487	76.56	68.1
woven-sweep-85	3.168e-05	16	384	4	0.003613	77.52	68.05
efficient-sweep-7	3.693e-05	16	384	4	0.0008038	76.67	68

Name	learning_rate	batch_size	max_length	epochs	weight_decay	f1	exact_match
azure-sweep-154	4.019e-05	32	384	4	0.001382	76.9	68
ethereal-sweep-124	7.754e-05	64	256	2	0.003582	77.06	67.95
revived-sweep-51	4.396e-05	32	384	3	0.0007941	77.11	67.95
efficient-sweep-106	3.15e-05	64	256	2	0.00349	76.76	67.86
confused-sweep-156	2.231e-05	16	384	3	5.865e-06	77.07	67.81
fragrant-sweep-99	2.836e-05	16	512	4	0.001861	77.05	67.76
morning-sweep-138	5.338e-05	64	384	2	1.88e-05	76.93	67.76
youthful-sweep-48	6.043e-05	64	256	4	5.549e-05	76.62	67.76
robust-sweep-94	6.194e-05	16	512	3	0.0001266	76.91	67.76
devout-sweep-28	7.257e-05	32	512	4	0.00148	76.71	67.71
youthful-sweep-108	6.698e-05	16	512	3	2.634e-05	76.87	67.71
kind-sweep-56	4.851e-05	64	384	4	5.25e-06	76.85	67.71
smooth-sweep-36	5.29e-05	32	384	3	0.00313	77.09	67.71
ancient-sweep-19	5.956e-05	16	256	3	0.001186	76.53	67.71
twilight-sweep-86	3.027e-05	16	384	3	0.00401	76.85	67.67
clear-sweep-136	6.593e-05	32	512	3	0.007995	76.55	67.67
fresh-sweep-87	3.755e-05	32	384	4	0.001073	77.06	67.67
wobbly-sweep-134	4.411e-05	16	512	4	1.566e-06	76.74	67.62
peach-sweep-75	2.576e-05	64	384	4	1.246e-06	77.03	67.62
noble-sweep-109	4.408e-05	64	256	3	0.006491	76.83	67.62
stellar-sweep-126	4.754e-05	32	512	4	2.343e-05	76.61	67.57
stoic-sweep-91	2.78e-05	32	384	4	0.001979	76.86	67.57
ethereal-sweep-30	2.112e-05	64	256	4	0.002299	76.85	67.57
polar-sweep-14	3.066e-05	32	512	4	0.0002198	76.97	67.57
revived-sweep-38	2.154e-05	16	384	3	3.476e-05	77.24	67.57
fanciful-sweep-102	4.232e-05	64	256	3	6.618e-05	76.59	67.52
rosy-sweep-2	7.159e-05	32	384	3	1.003e-05	76.81	67.52
dark-sweep-128	2.524e-05	32	384	3	1.696e-05	76.93	67.43
efficient-sweep-16	6.693e-05	16	512	3	0.0007753	76.68	67.43
eternal-sweep-10	3.162e-05	16	256	3	0.0004503	76.34	67.43
decent-sweep-157	2.74e-05	32	384	4	0.0005391	76.49	67.33
earnest-sweep-54	2.908e-05	64	384	4	9.275e-05	76.52	67.33
lyric-sweep-58	5.192e-05	32	512	3	1.469e-05	76.44	67.33
peach-sweep-78	5.553e-05	16	384	2	8.29e-06	76.91	67.33
deft-sweep-11	2.381e-05	16	256	2	0.0003328	76.59	67.33
fine-sweep-22	2.097e-05	32	384	4	3.532e-06	76.91	67.29
earnest-sweep-110	2.951e-05	32	256	3	0.002134	76.12	67.29
misunderstood-sweep-27	3.456e-05	16	384	3	5.398e-05	76.97	67.29

Name	learning_rate	batch_size	max_length	epochs	weight_decay	f1	exact_match
soft-sweep-52	2.495e-05	16	384	2	1.174e-06	76.7	67.29
youthful-sweep-151	2.171e-05	32	256	3	0.0007143	76.32	67.24
happy-sweep-123	3.058e-05	64	256	2	0.001733	76.37	67.24
generous-sweep-114	6.203e-05	16	512	2	4.706e-05	76.69	67.24
mild-sweep-24	4.787e-05	64	256	2	6.454e-06	76.67	67.24
fragrant-sweep-72	5.361e-05	16	512	3	2.116e-06	76.77	67.24
fearless-sweep-29	3.081e-05	32	384	3	0.0001146	76.52	67.19
flowing-sweep-103	6.361e-05	32	512	4	1.089e-05	76.39	67.19
upbeat-sweep-141	5.481e-05	64	384	2	1.822e-06	76.82	67.19
avid-sweep-50	6.336e-05	64	384	3	1.017e-05	76.42	67.19
sweet-sweep-45	3.097e-05	32	512	2	1.996e-06	76.81	67.1
hopeful-sweep-130	7.34e-05	64	512	3	1.084e-06	76.26	67.1
woven-sweep-44	6.916e-05	16	384	3	0.001399	76.58	67.05
crisp-sweep-142	4.606e-05	64	256	4	0.0001352	75.71	67.05
warm-sweep-68	5.957e-05	16	512	3	0.0008646	76.32	67.05
winter-sweep-66	3.885e-05	32	384	2	1.492e-06	76.5	67
noble-sweep-116	2.236e-05	16	512	3	0.0002619	76.5	67
zesty-sweep-131	4.091e-05	16	512	2	1.97e-05	76.34	66.95
autumn-sweep-95	2.413e-05	16	384	2	0.0002296	76.21	66.95
clear-sweep-96	6.103e-05	64	512	2	5.281e-06	76.32	66.9
balmy-sweep-104	4.332e-05	64	384	4	0.0006388	76.42	66.86
floral-sweep-46	2.354e-05	32	512	4	1.464e-05	75.87	66.81
splendid-sweep-5	2.914e-05	16	512	2	0.001494	76.19	66.76
northern-sweep-97	2.478e-05	64	384	3	6.022e-06	76.37	66.76
sandy-sweep-17	2.483e-05	16	512	2	4.81e-06	75.99	66.76
likely-sweep-122	2.816e-05	32	384	2	4.982e-06	75.84	66.76
exalted-sweep-77	5.125e-05	32	384	2	0.005132	76.5	66.71
decent-sweep-143	2.786e-05	32	384	2	0.0005984	75.93	66.71
dashing-sweep-81	7.087e-05	16	256	2	4.411e-05	76.11	66.71
magic-sweep-35	6.45e-05	64	512	2	0.0004633	76.07	66.52
blooming-sweep-32	2.032e-05	16	256	2	0.009832	75.51	66.48
lunar-sweep-62	2.028e-05	32	512	3	1.96e-05	76.47	66.48
divine-sweep-159	2.089e-05	16	256	3	8.891e-05	75.78	66.33
good-sweep-118	2.135e-05	64	512	4	5.323e-06	76.16	66.29
honest-sweep-21	3.386e-05	32	512	2	3.956e-05	75.59	66.24
peachy-sweep-42	6.048e-05	64	256	2	7.927e-05	75.62	66.1
efficient-sweep-83	3.935e-05	64	512	3	6.251e-06	75.6	66.1
pretty-sweep-34	2.046e-05	16	256	2	2.135e-05	75.98	66.1

Name	learning_rate	batch_size	max_length	epochs	weight_decay	f1	exact_match
vibrant-sweep-117	2.072e-05	16	512	2	0.0007143	75.16	65.95
sweepy-sweep-41	2.041e-05	16	512	2	0.001813	75.47	65.95
fancy-sweep-23	6.642e-05	16	512	2	0.0001729	75.54	65.9
usual-sweep-61	3.24e-05	64	512	4	3.502e-06	75.81	65.9
apricot-sweep-73	4.124e-05	64	512	2	7.781e-06	75.92	65.9
fast-sweep-69	2.807e-05	64	384	3	0.00524	75.54	65.81
icy-sweep-20	3.227e-05	16	512	2	0.001419	75.4	65.76
resilient-sweep-26	2.464e-05	32	512	2	0.00283	75.55	65.71
light-sweep-147	2.077e-05	16	384	2	2.516e-06	75.43	65.62
lucky-sweep-40	2.308e-05	64	512	4	3.797e-05	75.26	65.43
apricot-sweep-107	2.379e-05	32	512	4	3.506e-06	75.02	65.38
divine-sweep-8	3.066e-05	16	512	2	2.186e-05	75.22	65.38
proud-sweep-112	2.333e-05	64	384	3	1.398e-06	75.28	65.29
stilted-sweep-144	2.613e-05	64	512	2	6.565e-05	74.7	65.29
snowy-sweep-121	7.693e-05	32	512	2	0.007039	75.78	65.1
gentle-sweep-53	2.089e-05	16	512	2	0.0003838	74.97	65.05
clear-sweep-60	2.636e-05	16	384	2	8.179e-05	75.31	64.76
unique-sweep-63	2.326e-05	64	512	2	6.824e-05	73.76	63.43

In parallel, similar optimization sweeps were performed for the generative LLaMA-3.1 model, covering a comparable range of hyperparameters and assessed with the same performance metrics. The complete results are summarized in Table S3.

Table S3: Comprehensive hyperparameter sweep results for the LLaMA-3.1 model.

Name	learning_rate	lora_alpha	warmup_steps	lora_dropout	lora_r	eval_loss
true-sweep-63	0.0004063569634153	16	50	0.1	32	0.2080763578414917
silver-sweep-50	0.0002544253970567	32	50	0.1	32	0.2095046639442444
chocolate-sweep-57	0.0002571162244766	32	50	0.05	32	0.209723025560379
serene-sweep-35	0.0002232888321034	64	50	0.1	32	0.2110636383295059
silent-sweep-47	0.0004070488403967	32	50	0.1	32	0.2115794569253921
chocolate-sweep-3	0.0001904578050339	32	50	0.05	16	0.2123231440782547
vague-sweep-6	0.0004919676772358	32	50	0.05	16	0.2128503918647766
silent-sweep-49	0.0001842339784635	32	50	0.1	32	0.2130066901445388
swept-sweep-37	0.0003307450611416	16	50	0.05	32	0.2130289673805236
swept-sweep-60	0.0003323773800241	16	50	0.1	32	0.2140291482210159
vague-sweep-68	0.0002186189163885	16	50	0.05	16	0.2154598236083984

Name	learning_rate	lora_alpha	warmup_steps	lora_dropout	lora_r	eval_loss
swept-sweep-62	0.0003429219051927	16	50	0.1	32	0.2159692943096161
bold-sweep-14	0.0002414595427097	16	50	0.05	32	0.2166552245616912
bold-sweep-22	7.54191079091e-05	64	50	0.05	32	0.217771902680397
silver-sweep-34	0.0001938420549456	16	50	0.1	32	0.2188718020915985
polished-sweep-48	0.0001898971859555	32	100	0.1	32	0.2189073115587234
bold-sweep-27	0.0001692187342521	16	50	0.05	16	0.2189790308475494
bold-sweep-42	0.0001098238253028	32	50	0.1	16	0.2191964983940124
gentle-sweep-45	0.0001799318442723	32	100	0.1	32	0.2210767269134521
bright-sweep-51	0.0003056878666375	16	100	0.1	32	0.2211591899394989
silver-sweep-46	0.0002353862446085	32	100	0.1	32	0.2216407060623169
serene-sweep-67	0.0001612841469292	32	100	0.1	32	0.2239264249801635
silver-sweep-23	5.87263292831e-05	64	50	0.1	8	0.2244078665971756
gentle-sweep-24	0.000246939279104	32	100	0.1	32	0.2251448929309845
silver-sweep-59	0.0002643811445635	16	100	0.1	32	0.2255935966968536
silver-sweep-11	0.0001013930113244	64	100	0.1	16	0.2257167547941208
silver-sweep-13	0.0001051674682526	32	100	0.05	8	0.2259363085031509
silver-sweep-64	0.000395631929305	16	100	0.1	32	0.2260911166667938
silver-sweep-54	0.0003472935984313	32	100	0.1	32	0.226156622171402
swept-sweep-9	6.82416530007e-05	64	100	0.1	16	0.2263027727603912
polished-sweep-55	0.0002366221892568	16	100	0.1	32	0.2267626672983169
swept-sweep-28	8.94025400992e-05	32	100	0.05	16	0.2267723381519317
chocolate-sweep-44	0.000239014267215	32	100	0.1	32	0.2269353717565536
true-sweep-43	9.56955211357e-05	32	100	0.1	32	0.2287652045488357
bold-sweep-58	0.0002541598801366	32	100	0.05	16	0.2294087409973144
silent-sweep-20	0.0001088812048865	32	100	0.05	8	0.2298676520586013
polished-sweep-52	0.0002564000960092	32	100	0.05	16	0.230526328086853
silver-sweep-5	0.00015504881709942	16	100	0.05	32	0.2305799126625061
bold-sweep-15	5.60519504898e-05	64	100	0.05	32	0.2310710698366165
serene-sweep-18	0.0004462108232037	16	100	0.05	8	0.2314661741256714
true-sweep-33	0.0002161395678449	64	100	0.05	32	0.2321038842201233
chocolate-sweep-16	5.71701270232e-05	64	100	0.05	8	0.2327376455068588
silver-sweep-56	0.0003267147994963	32	100	0.05	32	0.2329990416765213
bold-sweep-61	0.0004854106904923	16	100	0.1	32	0.2340115904808044
silent-sweep-53	0.0003162470880254	64	100	0.1	32	0.2393635809421539
bright-sweep-30	0.0003863451918411	32	100	0.1	32	0.2401411980390548
true-sweep-32	3.75527794081e-05	64	50	0.05	32	0.2410923093557357
polished-sweep-25	0.0004531960661596	64	50	0.05	16	0.2431970536708832
serene-sweep-19	0.0004422233119035	32	100	0.1	16	0.243257001042366

Name	learning_rate	lora_alpha	warmup_steps	lora_dropout	lora_r	eval_loss
true-sweep-31	9.2315171524e-05	16	100	0.05	8	0.245845153927803
silent-sweep-69	4.80992759774e-05	32	50	0.05	32	0.2504854500293731
chocolate-sweep-10	4.59324875915e-05	32	50	0.1	32	0.2511868774890899
bright-sweep-40	0.0004907302216818	32	100	0.1	8	0.2545529007911682
chocolate-sweep-17	3.32528219845e-05	32	50	0.05	8	0.2665573060512543
bright-sweep-7	4.88238151288e-05	16	50	0.1	16	0.2676145732402801
vague-sweep-2	5.40060746126e-05	16	50	0.1	32	0.2699218690395355
silent-sweep-38	0.0004662040360856	64	100	0.1	16	0.2784733474254608
silver-sweep-66	1.52144183209e-05	64	100	0.1	16	0.2839950621128082
true-sweep-26	2.11201139499e-05	32	100	0.1	8	0.2872729301452636
bright-sweep-39	1.66509438931e-05	32	50	0.05	32	0.2922243773937225
vague-sweep-36	1.76224144675e-05	32	100	0.05	16	0.2924282550811767
serene-sweep-12	1.70216166913e-05	32	100	0.1	32	0.2935965061187744
gentle-sweep-21	2.69729171874e-05	16	100	0.1	16	0.2937659323215484
gentle-sweep-65	1.02768045354e-05	64	50	0.05	8	0.2938947379589081
swept-sweep-8	2.29072154952e-05	16	100	0.05	8	0.2978978455066681
vague-sweep-4	2.0139128479e-05	16	50	0.05	8	0.3017010986804962
silent-sweep-41	1.80213393471e-05	16	50	0.05	32	0.3105486035346985

SIV. Performance by Mechanical Property and Question Type

We provide additional, property-level performance analysis for the six models evaluated in this work: BERT-SQuAD, BERT-domain, XLNet-SQuAD, XLNet-domain, LLaMA-3.1-8B-Instruct, and LLaMA-domain. Results are stratified by mechanical property (ductility, fracture strength, ultimate tensile strength (UTS), yield strength, and Young's modulus) and by question type (entity lookup, numeric lookup, and property identification).

Figures S2-S4 summarize the performance using heatmaps, where each subplot corresponds to a single model, and each cell represents a specific property $\times$ question-type combination.

Figure S2 reports the EM score. The three domain models (BERT-domain, XLNet-

domain, and LLaMA-domain) consistently outperform the general-purpose baselines across nearly all properties and question types. LLaMA-domain achieves the highest EM values, particularly for property identification tasks.

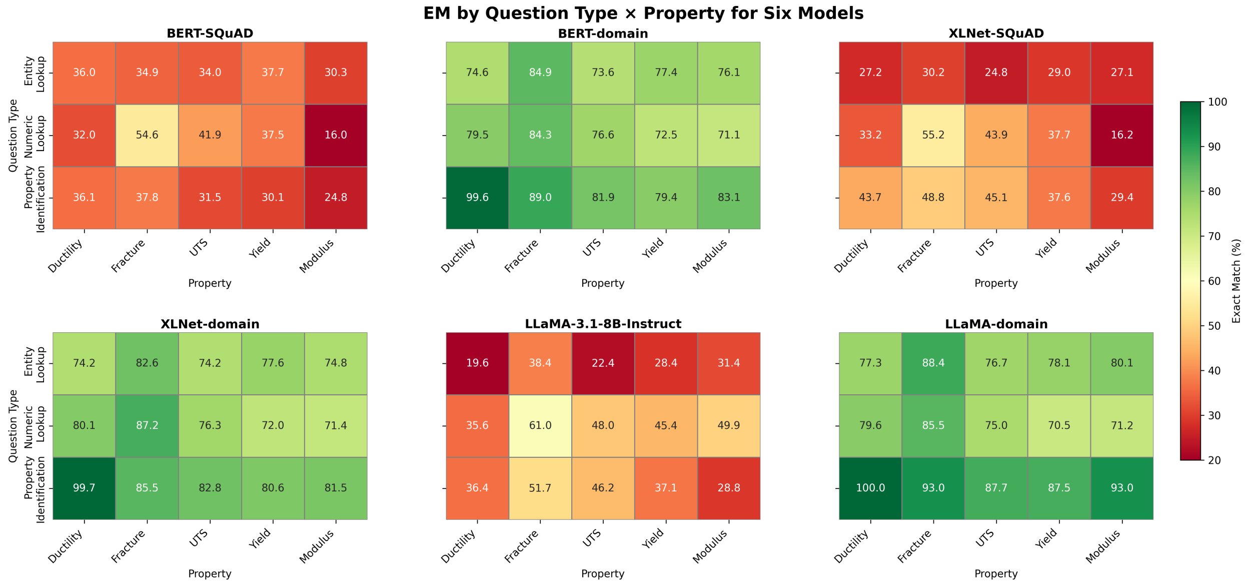

Figure S2: Exact Match performance by question type and mechanical property for six models. Each subplot corresponds to one model; cells show EM (%) for a given property–question-type combination.

Figure S3 reports the F1-score, which reflects partial lexical matching between predicted and reference answers. The trend broadly parallels the EM results: domain models show substantial gains over their baseline forms, and the LLaMA-domain model achieves the strongest overall performance.

Figure S4 reports the Expected Calibration Error (ECE), expressed as a percentage. Lower values indicate better alignment between model confidence and predictive accuracy. The extractive baselines (BERT-SQuAD and XLNet-SQuAD) exhibit the highest calibration error, particularly for numeric lookup questions involving Young's modulus and UTS. Calibration improves substantially for the three domain models, and LLaMA-domain yields the lowest overall ECE across properties and question types.

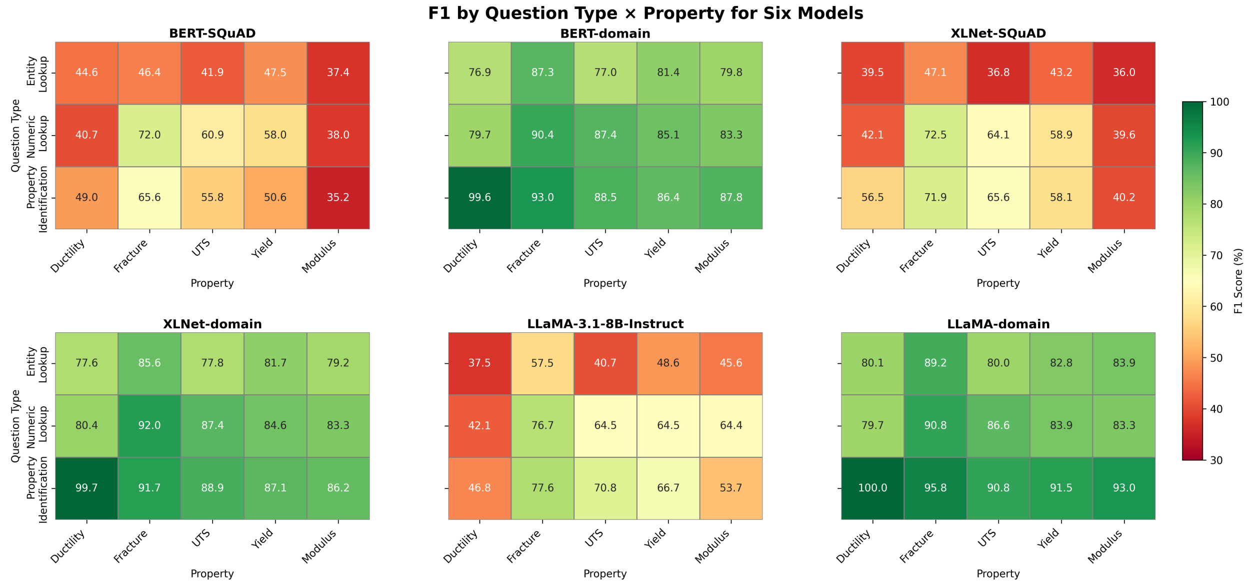

Figure S3: F1 performance by question type and mechanical property for six models. Each subplot corresponds to one model; cells show F1-score (%) for a given property–question-type combination.

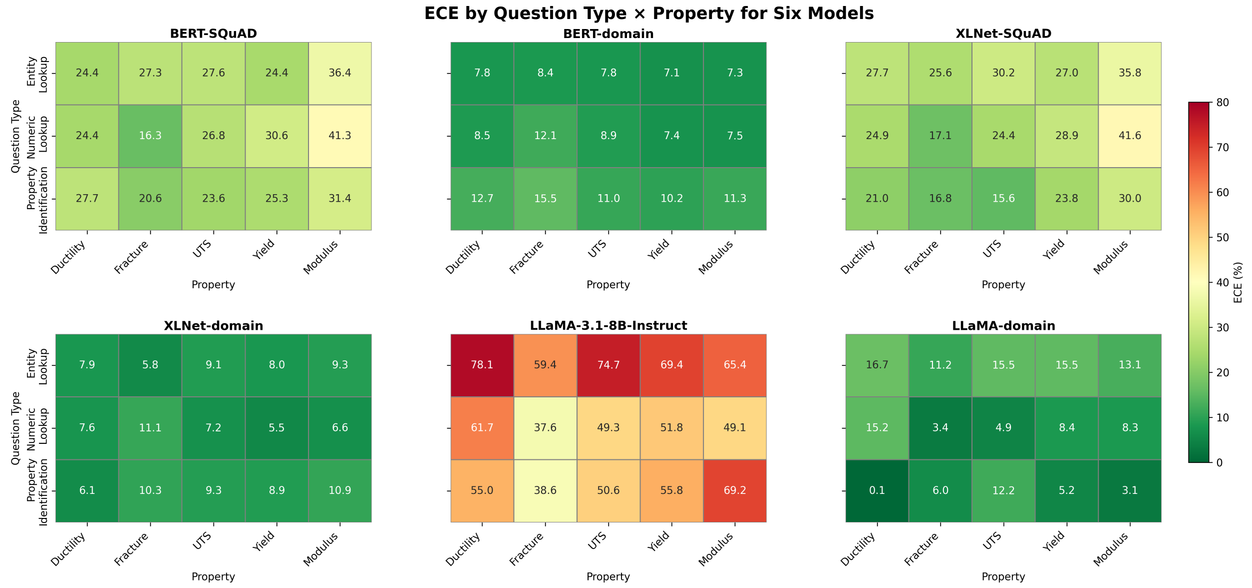

Figure S4: Expected Calibration Error by question type and mechanical property for six models. Each subplot corresponds to one model; cells show ECE (%) for a given property–question-type combination (lower is better).
